# Immunosuppressive role of BDNF in therapy‐induced neuroendocrine prostate cancer

**DOI:** 10.1002/1878-0261.13614

**Published:** 2024-02-21

**Authors:** Yen‐Nien Liu, Wei‐Yu Chen, Ming‐Kun Liu, Hsiu‐Lien Yeh, Wei‐Hao Chen, Kuo‐Ching Jiang, Han‐Ru Li, Zi‐Qing Chen, Wan‐Hsin Wang, Wassim Abou‐Kheir, Yu‐Ching Wen

**Affiliations:** ^1^ Graduate Institute of Cancer Biology and Drug Discovery, College of Medical Science and Technology Taipei Medical University Taiwan; ^2^ Department of Pathology, Wan Fang Hospital Taipei Medical University Taiwan; ^3^ Department of Pathology, School of Medicine, College of Medicine Taipei Medical University Taiwan; ^4^ Division of Clinical Pharmacy, School of Pharmacy Taipei Medical University Taiwan; ^5^ Department of Anatomy, Cell Biology and Physiological Sciences Faculty of Medicine American University of Beirut Lebanon; ^6^ Department of Urology, Wan Fang Hospital Taipei Medical University Taiwan; ^7^ Department of Urology, School of Medicine, College of Medicine Taipei Medical University Taiwan; ^8^ TMU Research Center of Urology and Kidney Taipei Medical University Taiwan

**Keywords:** androgen deprivation therapy, brain‐derived neurotrophic factor, leukemia inhibitory factor, neuroendocrine differentiation, prostate cancer, tumor microenvironment

## Abstract

Prostate stromal cells play a crucial role in the promotion of tumor growth and immune evasion in the tumor microenvironment (TME) through intricate molecular alterations in their interaction with prostate cancer (PCa) cells. While the impact of these cells on establishing an immunosuppressive response and influencing PCa aggressiveness remains incompletely understood. Our study shows that the activation of the leukemia inhibitory factor (LIF)/LIF receptor (LIFR) pathway in both prostate tumor and stromal cells, following androgen deprivation therapy (ADT), leads to the development of an immunosuppressive TME. Activation of LIF/LIFR signaling in PCa cells induces neuroendocrine differentiation (NED) and upregulates immune checkpoint expression. Inhibition of LIF/LIFR attenuates these effects, underscoring the crucial role of LIF/LIFR in linking NED to immunosuppression. Prostate stromal cells expressing LIFR contribute to NED and immunosuppressive marker abundance in PCa cells, while LIFR knockdown in prostate stromal cells reverses these effects. ADT‐driven LIF/LIFR signaling induces brain‐derived neurotrophic factor (BDNF) expression, which, in turn, promotes NED, aggressiveness, and immune evasion in PCa cells. Clinical analyses demonstrate elevated BDNF levels in metastatic castration‐resistant PCa (CRPC) and a positive correlation with programmed death‐ligand 1 (PDL1) and immunosuppressive signatures. This study shows that the crosstalk between PCa cells and prostate stromal cells enhances LIF/LIFR signaling, contributing to an immunosuppressive TME and NED in PCa cells through the upregulation of BDNF.

## Graphical abstract

Activation of LIF/LIFR/STAT3 signaling in the prostate cancer tumor microenvironment triggers BDNF production, linked to heightened *PDL1* expression and neuroendocrine differentiation in prostate cancer cells. This pathway is crucial in stromal cells for regulating immune suppression and checkpoint responses, suggesting potential prognostic markers and therapies to combat immune evasion and neuroendocrine differentiation in prostate cancer.

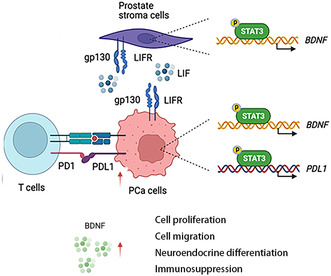

**Abbreviations**
ADT: androgen deprivation therapy
ANOVA: analysis of variance
BDNF: brain‐derived neurotrophic factor
BPH: benign prostatic hyperplasia
CAFs: cancer‐associated fibroblasts
CHGA: chromogranin A
CM: conditioned medium
CRPC: castration‐resistant prostate cancer
CSS: charcoal‐stripped serum
DEGs: differentially expressed genes
ELISA: enzyme‐linked immunosorbent assay
ENO2: enolase 2
ERK: extracellular signal‐regulated kinase
EV: empty vector
FACS: fluorescence‐activated cell sorting
GSEA: gene set enrichment analysis
HPEC: human prostate epithelial cells
IF: immunofluorescence
LIF: leukemia inhibitory factor
LIFR: leukemia inhibitory factor receptor
MAPK: mitogen‐activated protein kinase
MFI: median fluorescence intensity
NANOG: Nanog homeobox
NED: neuroendocrine differentiation
NEPC: neuroendocrine prostate cancer
PBS: phosphate‐buffered saline
PCa: prostate cancer
PD1: programmed cell death protein 1
PDL1: programmed death‐ligand 1
PI3K: phosphatidylinositol 3‐kinase
RT‐qPCR: reverse‐transcription‐quantitative polymerase chain reaction
SEM: standard error of the mean
SOX2: SRY‐box 2
STAT: signal transducer and activator of transcription
SYP: synaptophysin
TCGA: The Cancer Genome Atlas
TGF‐ß: transforming growth factor ß
TME: tumor microenvironment

## 1 Introduction

Prostate cancer (PCa) exhibits high heterogeneity, where genetic mutations and alterations in key pathways contribute to the development of advanced PCa [1]. Activation of the androgen receptor (AR) by androgens regulates normal prostate cell growth and function [1]. However, dysregulation of the AR pathway is a vital driver in the development of PCa, including the transition to castration‐resistant PCa (CRPC) [2]. CRPC primarily represents a stage of prostate adenocarcinoma where the disease progresses despite the application of androgen deprivation therapy (ADT) [2]. Prolonged ADT applies selective pressure on cancer cells, potentially leading to the emergence of a neuroendocrine phenotype, a phenomenon known as neuroendocrine differentiation (NED). Neuroendocrine PCa (NEPC) displays resistance to therapy and immune evasion mechanisms, which contribute to its insensitivity to immunotherapeutic approaches [3]. The tumor microenvironment (TME) in therapy‐resistant PCa can be immunosuppressive, characterized by immune checkpoint molecules and infiltration of immunosuppressive cell populations [4, 5, 6]. These factors diminish antitumor immune responses and promote tumor growth and progression.

Programmed death‐ligand 1 (PDL1), a critical immune checkpoint molecule, plays a crucial role in suppressing antitumor immune responses by interacting with its receptor, programmed cell death protein 1 (PD1), in immune cells [7]. Overexpression of PDL1 was associated with immune escape and poor prognoses in various cancers, including PCa [8]. Therefore, understanding the mechanisms underlying the regulation of PDL1 is of significant interest for developing effective therapeutic strategies. Several pathways have been implicated in regulating PDL1 expression [9, 10, 11]. Activation of the phosphatidylinositol 3‐kinase (PI3K)/Akt/mammalian target of rapamycin (mTOR) pathway, which is frequently dysregulated in cancer, was linked to increased PDL1 expression [12]. Similarly, activation of the mitogen‐activated protein kinase (MAPK)/extracellular signal‐regulated kinase (ERK) pathway, often associated with cell proliferation and survival, was also implicated in PDL1 upregulation [13]. Understanding the upregulation of immune checkpoint protein expression through various signaling pathways and their interactions with the TME is critical for the development of effective immunotherapies and combination strategies.

Leukemia inhibitory factor (LIF), a cytokine belonging to the interleukin (IL)‐6 family, influences various cellular processes, including inflammation, differentiation, and survival [14]. LIF binds to LIF receptor (LIFR), a heterodimer composed of ligand‐binding subunit (LIFRβ) and signal‐transducing subunit (GP130), activating downstream pathways, including Janus kinase (JAK)/signal transducer and activator of transcription (STAT) [15]. The activation of LIF/LIFR signaling has been implicated in promoting aggressiveness features in PCa cells [16]. LIF signaling activates transcription factors and pathways associated with NED [17]. Furthermore, the LIF/LIFR signaling pathway has been identified as a potential regulator of immune checkpoints and the TME in various cancer types, including PCa [18]. However, the specific downstream modulator of this signaling pathway remains unclear.

Brain‐derived neurotrophic factor (BDNF), belonging to the neurotrophin family, supports the growth, survival, and function of neurons [19]. Increased BDNF expression correlates with enhanced cell proliferation, resistance to apoptosis, and tumor progression, while also stimulating endothelial cell proliferation and migration, contributing to the development of a vascular network that supports tumor growth [20]. In PCa, BDNF promotes cell survival and proliferation through activation of PI3K/AKT and MAPK/ERK pathways [21]. However, the association of BDNF induction with NEPC development and its regulation by LIF/LIFR signaling in PCa after ADT remain unclear. Additionally, the relationship between BDNF abundance and immunosuppression in the PCa TME is not well defined.

The prostate stroma, comprising supportive connective tissue and cells around the prostate gland, can secrete factors suppressing immune responses, creating an immunosuppressive environment facilitating tumor cells' evasion of immune surveillance [22, 23]. We aimed to investigate the impact of LIF/LIFR signaling on the expressions of immune checkpoint molecules and BDNF in the PCa TME, exploring its influence on interactions between tumor cells and prostate stromal cells. Additionally, the potential clinical implications of BDNF as a biomarker for predicting immunosuppressive phenotypes and treatment responses in PCa patients were investigated. The analysis of the relationship between BDNF levels in PCa patients and BDNF‐driven NED could offer valuable insights into developing non‐invasive prognostic biomarkers and identifying potential therapeutic targets.

## 2 Materials and methods

### 2.1 Cell culture, reagents, and constructs

Normal human prostate epithelial cells (HPrEC, RRID: CVCL_V626) were obtained from ATCC and cultured in prostate epithelial cell basal medium (PrEBM, Lonza, Basel, Switzerland, CC‐3166). Adenocarcinoma PCa cells (LNCaP (C‐33, RRID: CVCL_0395) and PC3, RRID: CVCL_0035) were obtained from ATCC and cultured in RPMI‐1640 medium (ThermoFisher Scientific, Waltham, MA, USA, 11875‐085) supplemented with 10% fetal bovine serum (FBS; EMD Millipore, Burlington, MA, USA, TMS‐013‐BKR) and 1% penicillin. NEPC‐like cells (LASCPC01, RRID: CVCL_UE17) were obtained from ATCC and cultured in modified HITES medium, supplemented with 10 nm hydrocortisone (Sigma‐Aldrich, St. Louis, Missouri, USA, H0888), 0.01 mg·mL^−1^ transferrin (Sigma‐Aldrich), 4 mm l‐glutamine (Invitrogen, Carlsbad, CA, USA), 5% FBS, and 1% penicillin. Enzalutamide/MDV3100‐resistant cells (LNCaP‐MDVR) were derived from LNCaP cells that had developed resistance to 20 μm MDV3100 over 6 months. All cell lines have been authenticated within the past 3 years through short tandem repeat (STR) profiling. For conditioned medium (CM) treatment, human prostate stroma cells (WPMY‐1, RRID: CVCL_3814) obtained from ATCC were counted and seeded in 10‐cm plates at 3 × 10^6^ cells·mL^−1^ in 10 mL of serum‐free RPMI medium. Cells were then maintained in a 37 °C, 5% CO_2_ incubator. After 24 h, CM was collected and centrifuged at 1000 ***g*** for 5 min to remove particulates. LNCaP cells were cultured in a mixture of different percentages of WPMY‐1 cell CM (0%, 15%, or 50%) and standard LNCaP complete medium for the experiment. The human monocyte cell line THP‐1 (RRID: CVCL_0006) was used for culture in 150 nm phorbol 12‐myristate 13‐acetate (PMA, Sigma, P8139)‐containing medium. The PMA‐treated THP‐1 cells were subsequently cultured in CM collected from LNCaP cells expressing control or BDNF‐KD, in the presence or absence of ADT. Macrophage polarization assessment was determined by characterizing macrophages for M2 polarization markers expression using RT‐qPCR. All cell lines underwent regular checks for mycoplasma contamination using a Mycoplasma PCR Detection Kit (Omicsbio, New Taipei City, Taiwan, G238) within the last 6 months. To mimic ADT, cells were cultured in RPMI‐1640 medium containing 10% charcoal‐stripped serum (CSS, ThermoFisher, 12676‐029) under standard culture conditions. The LIF recombinant protein was used at a concentration of 100 ng·mL^−1^ for indicated times. An LIF inhibitor (EC330, MedChemExpress, Monmouth Junction, NJ, USA, HY‐100949) was administered at concentrations of 10 or 35 nm for 48 h. To achieve constitutive *LIF* overexpression, a pCDH‐CMV‐MCS‐EF1‐Neo vector (System Biosciences) encoding *LIF* full‐length complementary (c)DNA was used, with an empty vector (EV) serving as a control. To knockdown (KD) BDNF expression, cells were infected with a recombinant lentivirus carrying a human *LIFR* short hairpin (sh)RNA vector, and the Luciferase (Luc) shRNA vector was used as a non‐target control. To construct regulatory sequence reporters, the pGreenFire reporter (System Biosciences, Palo Alto, CA, USA) was used, and a DNA sequence analysis verified all constructs. Primers used for generating these constructs are listed in Table S1.

### 2.2 Reverse‐transcription (RT)‐quantitative polymerase chain reaction (qPCR)

The mRNA isolation was carried out using an RNeasy Midi Kit (Qiagen, Hilden, Germany, 74004), and 1 μg of total mRNA was utilized for RT using an iScriptTM cDNA Synthesis Kit (Bio‐Rad, Hercules, CA, USA, 1708890). For amplification, iTaq Universal SYBR Green Supermix (Bio‐Rad, 1725120) was employed in a thermocycler. The amplification protocol involved initial incubation at 95 °C for 10 min, followed by 40 cycles at 95 °C for 15 s and 60 °C for 1 min. All reactions were performed in triplicate, and expression levels were normalized to human 18S ribosomal (r)RNA. Primer pairs used in this process are specified in Table S2.

### 2.3 Western blot analysis

Protein samples were lysed from cells using 200 μL of RIPA buffer (ThermoFisher Scientific, 8900) supplemented with a protease inhibitor (Roche, Basel, Switzerland, 11697498001) and a phosphatase inhibitor cocktail (Roche, 4906845001). The protein concentration in each sample was determined using the Bradford reagent (Bio‐Rad, 5000006), and samples were subsequently separated by sodium dodecylsulfate polyacrylamide gel electrophoresis (SDS/PAGE). Following separation, proteins were transferred onto either polyvinylidene difluoride (PVDF) or nitrocellulose membranes (ThermoFisher Scientific). Membranes were then blocked with a solution of 5% bovine serum albumin (BSA) in a Tris‐based buffer containing 0.1% Tween‐20 for 1 h. Enhanced chemiluminescence (ECL) with a western blotting detection reagent (Millipore, WBULS0100) was used to visualize protein bands. Specific information about the antibodies utilized for western blotting can be found in Table S3. Protein intensities were measured by imagej software (LOCI/NIH, Bethesda, MD, USA) and determined from three independent experiments.

### 2.4 Immunofluorescence (IF) staining

For IF staining of LIFR, PDL1, and BDNF in LNCaP cells expressing either control or LIFR shRNA at a density of 5 × 10^4^ cells per well were seeded onto Millicell EZ slide multi‐chamber slides (Merck Millipore) and cultured in medium containing 5% CSS and treated with 100 ng·mL^−1^ of the LIF protein for 48 h. Following treatment, cells were sequentially fixed with 4% paraformaldehyde in phosphate‐buffered saline (PBS) for 10 min and permeabilized with 0.1% Triton‐X100 in PBS for 5 min. Fixed cells were then blocked using 5% BSA in 0.1% PBST (PBS with Tween‐20) for 1 h and stained with a LIFR antibody (1 : 100; ab101228; Abcam, Cambridge, UK), PDL1 antibody (1 : 100; 66248‐1; Proteintech, Rosemont, IL, USA), and BDNF (1 : 100; ab108319; Abcam) at 4 °C overnight. Samples stained with the primary antibody were subsequently labeled with a donkey anti‐rabbit IgG highly cross‐adsorbed secondary antibody (Alexa Fluor™ 488, A‐21206; Thermo Fisher Scientific) and a donkey anti‐mouse IgG highly cross‐adsorbed secondary antibody (Alexa Fluor™ 647, A‐31571; Thermo Fisher Scientific) for 1 h and mounted using DAPI‐Fluoromount‐G™ mounting medium (Electron Microscopy Sciences, Hatfield, PA, USA). Images were captured using a fluorescence microscope (IX73, Olympus, Tokyo, Japan).

### 2.5 Proliferation assay

For cell proliferation assays, LNCaP cells were seeded in 96‐well plates at a density of 3000 cells per well. CM treatments were established by combining regular LNCaP complete medium with 50% CM from WPMY‐1 cells expressing a control or LIFR shRNA vector. The proliferation rate was monitored every 24 h for 5 days. Cells were stained daily with 0.5% crystal violet for 15 min, followed by four washes with distilled water and subsequent drying. Before the measurement, crystal violet was dissolved by adding 100 μL of 50% ethanol containing 0.1 m sodium citrate to each well, with gentle shaking to ensure complete dissolution. Absorbances at two wavelengths, 540 and 405 nm, were measured with a microplate reader. Multiple wells were used at each time point, and average values were recorded for analysis.

### 2.6 Sphere formation assay

LNCaP cells at a density of 500 cells per well in a complete medium were combined with the desired amount of standard Matrigel matrix (Corning, NY, USA, 354234), and the mixture was added to the bottom edge of a six‐well plate. After incubation at room temperature for 15 min, the mixture was cultured with 50% CM collected from WPMY‐1 cells expressing either LIFR‐KD or a control vector and incubated for 7 days. For studies on LIF overexpression in BDNF‐KD cells, LNCaP cells were stably transfected with either Luc or BDNF shRNA. This was followed by the expression of either an empty vector (EV) or LIF cDNA vector, and the cells were then cultured in complete medium for 7 days. Tumor spheroids in each well were observed and photographed using a phase‐contrast microscope (Olympus), and their number and size were recorded.

### 2.7 Migration and invasion through Matrigel assays

LNCaP cells were cultured with 50% CM collected from WPMY‐1 cells expressing either a LIFR‐KD or control vector. For LIF overexpression combined with BDNF‐KD, LNCaP cells were stably transfected with control or BDNF shRNA vector, followed by control or LIF cDNA vector overexpression. For the migration assay, 3 × 10^4^ cells per well were suspended in serum‐free medium and added to 24‐well Boyden chambers (8‐μm pore size), and chambers were placed in a 24‐well culture plate. For the invasion assay, Boyden chambers were precoated with 200 μg·mL^−1^ Matrigel matrix (Corning, 354234). The lower chamber was loaded with 600 μL of complete medium without the use of proliferation inhibitors, and the entire plate was incubated for 12 h under standard cell culture conditions at 37 °C with 5% CO_2_ to facilitate invasion. After incubation, Matrigel‐coated Boyden chambers were fixed with methanol for 5 min and stained with 0.5% crystal violet for 15 min. Non‐invaded cells were removed from the chamber using a cotton swab, followed by washing with distilled water, while invaded cells remained on the underside of the membrane. The chamber was air‐dried at room temperature, and triplicate images of migrating or invading cells on the underlying membrane were captured using a phase‐contrast microscope (Olympus).

### 2.8 Tumorigenicity assays in mice

Animal experimental procedures in this study adhered to the *Guidelines for Care and Use of Laboratory Animals* by the Council of Agriculture, Executive Yuan, Taiwan, and received approval from the Taipei Medical University Institutional Animal Care and Use Committee under approval ID: LAC‐2021‐0481. This approval included the experimental protocols and the overarching research objectives, methodologies, and data‐handling procedures, ensuring compliance with ethical standards and regulations for animal welfare. In a double‐blind fashion, four 6‐week‐old male nude mice (BALB/c Nude:CAnN.Cg‐Foxn1nu/CrlNarl) were obtained from Academia Sinica (Taipei, Taiwan). The mice were housed under controlled conditions, including temperature (20–26 °C), humidity (30–70%), and lighting (12‐h light/12‐h dark cycles), to maintain the animals' circadian rhythms and comfort. Each experimental group of mice was subcutaneously injected with 2 × 10^6^ LNCaP/EV + shLuc, LNCaP/LIF + shLuc, or LNCaP/LIF + shBDNF cells into the right flank. Cells were suspended in 100 μL of a mixture containing 50% Matrigel matrix and 50% complete medium. Tumor sizes and mouse body weights were measured weekly for 8 weeks. The tumor volume (*V*) was calculated using the formula *V* = 0.5236 × *H* × *W* × *L*, where *H*, *W*, and *L* represent the tumor's height, width, and length, respectively.

### 2.9 Immunohistochemical (IHC) staining

For IHC staining for LIFR, BDNF, PDL1, ENO2, and KI67, tumors were harvested 2 months later from mice subcutaneously injected with LNCaP/EV + shLuc, LNCaP/LIF + shLuc, or LNCaP/LIF + shBDNF cells. Before performing IHC staining, tumor slices underwent deparaffinization, rehydration, and heat treatment. Subsequently, specific primary antibodies were used for staining and targeting proteins such as LIFR, BDNF, PDL1, ENO2, and KI67, as outlined in Table S4. The PCa tissue microarray (TMA) for BDNF IHC staining was purchased from TissueArray.Com LLC (Derwood, MD, USA) (*n* = 40, PR483d, MD, USA). The study, including all associated protocols, received approval from the Taipei Medical University Joint Institutional Review Board under approval no. N202201094, ensuring comprehensive ethical oversight not just for the protocols but for the entire research project. A washing solution composed of Tris‐buffered saline (TBS) buffer with 0.1% Triton X‐100, conjugated with avidin, and colored with the 3.3′‐diaminobenzidine reagent was applied. After the washing steps, slices were stained with a secondary antibody, allowed to dry, and mounted using glycerol. Ten bright‐field microscopic images of IHC‐stained sections were captured from each core using a phase‐contrast microscope at 200× magnification (Olympus IX73) for the histomorphometric analysis of tissue sections. The intensity of the target proteins was categorized as 0 (negative), 1+ (weakly positive), 2+ (moderately positive), or 3+ (strongly positive). To calculate the intensity scoring values ranging from 0 to 300, the following formula was used: (1 × percent of 1+ cells) + (2 × percent of 2+ cells) + (3 × percent of 3+ cells). The *P*‐values were calculated by a Chi‐squared test performed using spss statistical 18.0 software (IBM, Armonk, NY, USA).

### 2.10 Chromatin immunoprecipitation (ChIP) assay and ChIP‐sequencing (Seq) data analysis

The ChIP assay was performed using the EZ‐Magna ChIP™ IP Kit (Sigma‐Aldrich, 17‐10086) according to the manufacturer's manual. Human stromal WPMY‐1 cells or LNCaP cells were treated with either PBS or 100 ng·mL^−1^ of the LIF recombinant protein, followed by treatment with 0.1 and 35 nm EC330 for 48 h. After the treatment, cells were fixed with 1% paraformaldehyde in a complete medium for 10 min, and fixation was stopped by incubation with 125 mm glycine buffer for 5 min. Fixed cells were then washed with cold PBS containing protease and phosphatase inhibitors and collected by scraping into PBS buffer to obtain cell debris. Sonication (Qsonica, Newtown, CT, USA) was used with assay buffer from the kit to generate chromatin fragments of approximately 150 bp. Chromatin protein complexes were immunoprecipitated using 10 ng of an anti‐p‐STAT3 antibody (Sigma‐Aldrich, 06‐680), anti‐acetyl histone H3 antibody (positive control, Novus, Centennial, CO, USA, NB300‐221), or normal rabbit immunoglobulin G (IgG) as a negative control (Santa Cruz, Dallas, Texas, USA, sc‐2027), along with protein A‐coated magnetic beads. Chromatin was then released from the complexes by proteinase K (Sigma‐Aldrich, 124568) after heat inactivation and identified using an RT‐qPCR. ChIP antibodies and qPCR primers used in the experiment are listed in Table S5. In this study, we performed a ChIP‐sequencing data analysis to investigate the binding ability of STAT3 to the *BDNF* and *PDL1* genes. ChIP‐sequencing data were retrieved from the Gene Expression Omnibus (GEO) database with accession numbers GSM2752894 and GSM2752900 [24]. The Genome Browser, a powerful tool offered by the Genomics Institute at the University of California, Santa Cruz (UCSC) (CA, USA), was employed to analyze these data.

### 2.11 Promoter reporter assay

The STAT3 response element (SRE) regions of the human *BDNF* and *PDL1* genes were identified at specific chromosomal locations (*BDNF*/SRE1: 11:27704858, *BDNF*/SRE2: 11:27724660, and *BDNF*/SRE3: 11:27727406; *PDL1*/SRE1: 9:5447243, *PDL1*/SRE2: 9:5448096, and *PDL1*/SRE3: 9:5449792) in the GRCh38 genome. These regulatory sequences were incorporated into a GFP reporter vector (pGreenFire1‐ISRE Lentivector; System Biosciences) using the Clone‐it Enzyme‐Free Lentivector Kit (System Biosciences). A Site‐Directed Mutagenesis System kit (Invitrogen) was employed for the human BDNF and PDL1 genes to generate SRE mutant constructs. Wild‐type (WT) or mutant (M) GFP‐reporter constructs were transiently transfected into WPMY‐1 or LNCaP cells using X‐tremeGENE HP DNA Transfection Reagent (Sigma‐Aldrich, 6366236001). Transfected cells were treated with either PBS or 100 ng·mL^−1^ LIF protein, followed by treatment with 10 or 35 nm EC330 for 48 h. Promoter activity was assessed using fluorescence‐activated cell sorting (FACS, BD Biosciences, San Jose, CA, USA) by measuring relative median fluorescence intensity (MFI) GFP values normalized to the vehicle control. Three independent experiments were conducted with triplicate samples. Primers used to generate SRE‐M constructs of the human *BDNF* and *PDL1* genes are given in Table S1.

### 2.12 Enzyme‐linked immunosorbent assay (ELISA)

Serum samples from patients with benign prostatic hyperplasia (BPH, 20 samples), hormone‐sensitive PCa (HSPC, 20 samples), and CRPC (16 samples) were obtained from Taipei Medical University‐Affiliated Hospital (Taipei, Taiwan). Informed consent was obtained in writing from all participants, ensuring their access to information while maintaining privacy and confidentiality. The study methodologies, characterized by scientific validity, were reviewed and approved by the Taipei Medical University Joint Institutional Review Board (from January 2021 to December 2023), and conformed to the standards set by the Declaration of Helsinki. After blood collection, whole‐blood tubes were allowed to clot for 30 min, and serum was centrifuged at 1000 ***g*** for 20 min to remove any clotting particles. Cell culture supernatants were also centrifuged at 1000 ***g*** for 20 min to eliminate particles. The obtained serum samples were aliquoted and stored at −80 °C until further use. BDNF levels were measured with a human BDNF ELISA kit (MyBiosource, San Diego, CA, US, MBS824804) following the manufacturer's instructions. The optical density (OD) of each standard, control, and sample was determined by subtracting the average zero‐standard OD from the average of duplicate readings. A standard curve was generated using software capable of constructing a four‐parameter logistic fit curve, and BDNF levels in the samples were calculated based on this curve.

### 2.13 Dataset analysis

Expression data from human PCa datasets of The Cancer Genome Atlas (TCGA) [25] were log_2_‐normalized. Gene set enrichment analysis (gsea) software [26] was utilized, and the upregulated gene signatures of transforming growth factor (TGF)‐ß, interleukin (IL)‐10, and IL‐6 in cytokine‐responsive (Biocarta or Abbud) stromal cells from hematopoietic stem cells (HSCs) (Durand), stromal cells from metastatic stromal cells (Sung), NEPC‐responsive, and neuronal developmental responsiveness (KEGG, GO, and REACTOME) datasets were obtained from the gene expression omnibus (GEO). These gene signatures were then used to assess correlations of *LIFR* and *BDNF* with mRNA levels. The GSEA program calculated the normalized enrichment score (NES) and false discovery rate (FDR). Cutoff values were predetermined using one‐half the number of patients from the GSEAs. Negative and positive correlations between LIFR expression and the number of immune cell infiltrates in PCa were analyzed using PRAD databases provided by TIMER 2.0 [27]. Immune cell types assessed included cancer‐associated fibroblasts (CAFs), M2 macrophages, regulatory T (Treg) cells, M1 macrophages, CD8^+^ T‐cells, CD4^+^ type 1 helper T‐cells, myeloid‐derived suppressor cells, myeloid dendritic cells activated, natural killer (NK) cells, and NK T‐cells. Spearman's correlations and purity adjustments were applied for the analysis. Immune infiltration was evaluated using various algorithms, including the TIDE, CIBERSORT, CIBERSORT‐ABS, XCELL, and QUANTISEQ algorithms provided by TIMER 2.0. The cohorts represent Kaplan–Meier plots generated using an advanced online survival analysis tool that facilitates real‐time calculations. This tool stands out for its meticulous curation of a database, which involves manual compilation of gene expression data and relapse‐free as well as overall survival information. The database is efficiently managed by a PostgreSQL server that consistently monitors and expands repositories and clinical data, integrating gene expression and clinical information. Survival charts were generated using the online gepia tool, which included 275 PCa samples from TCGA and GTEx projects [28]. Our analysis focused on exploring the role of the *BDNF* gene in disease‐free survival within the context of PCa. Hazard ratios (HRs) with 95% confidence intervals (CIs) and log‐rank *P*‐values were employed to determine the significance of BDNF expression in relation to these cancer cases. A Kaplan–Meier plotter analysis was performed to assess the overall survival rates of patients in both the high‐BDNF expression group (treated with anti‐PD1 or anti‐PDL1) and slightly low‐BDNF expression group (no treatment) provided by the Kaplan–Meier plotter [29]. A log‐rank test was used to determine the statistical significance of survival differences between the two groups for immunotherapy. The analysis was performed using a selected probe set for BDNF expression levels, with 21, 50, and 56 cutoff values. The expression range of the BDNF probe set varied from 1 to 2118 or 3 to 2060, as analyzed by the Kaplan–Meier plotter.

### 2.14 RNA‐Seq analysis

Transcriptome analyses of both parental LNCaP cells and LNCaP‐MDVR cells were conducted using BioTools (Xizhi, New Taipei, Taiwan), following the complete protocol provided by BioTools. The comprehensive transcriptome analysis process encompassed RNA extraction and sequencing, data filtration and mapping, and an ontology analysis. To elaborate, mRNA and cDNA libraries were prepared using KAPA mRNA HyperPrep Kits (Roche) and sequenced using the NovaSeqTM 6000 sequencing system (Illumina, San Diego, CA, USA) in 300~400‐bp paired‐end reads. The quality of the raw reads was checked using fastqc and multiqc during data modulation. Subsequently, raw paired‐end reads were trimmed using trimommatic v0.38 (leading: 3; trailing: 3; sliding window: 4:15; Minlen: 30), and contamination was detected using diamond [30, 31, 32]. Clean reads were then mapped to the reference genome (*Homo sapiens* GRCh38) using hisat2 v2.1.0, and featurecount v1.6.0 was employed to map read counts to individual genes. Read counts were normalized to relative log expressions using the deseq2 v1.22.1 package in r. All parameters and codes from each r package followed default settings, except for those mentioned earlier. Differentially expressed genes (DEGs) were determined using deseq2, setting the threshold of significance to log[fold change of > 2] and an adjusted *P*‐value of < 0.05, based on Benjamini and Hochberg's approach [33]. A volcano plot was constructed with the log2[fold change] on the *x*‐axis and the negative logarithm (base 10) of the *P*‐value (or adjusted *p*‐value) on the *y*‐axis. This representation enables better visualization of significant genes and reduces the impact of extreme *P*‐values. Upregulated genes are depicted on the right side of the plot, while downregulated genes are on the left side. Significant genes with low *P*‐values are displayed toward the top of the plot.

### 2.15 Statistical analyses

graphpad prism software version 8.0 (GraphPad Software, Inc., San Diego, CA, USA) was utilized for graph construction, and results are depicted as the mean ± standard error of the mean (SEM). Statistical significance between groups was assessed using a one‐way analysis of variance (ANOVA), two‐way ANOVA, two‐tailed *t*‐test, and Bonferroni *post‐hoc* test. A paired two‐tailed Student's *t*‐test was employed to compare the IHC staining of tissue samples. A *P*‐value of < 0.05 was considered statistically significant. All experiments were independently repeated at least three times.

## 3 Results

### 3.1 LIF/LIFR signaling drives NED, which is associated with the immunosuppressive response in PCa cells

**Fig. 1:**
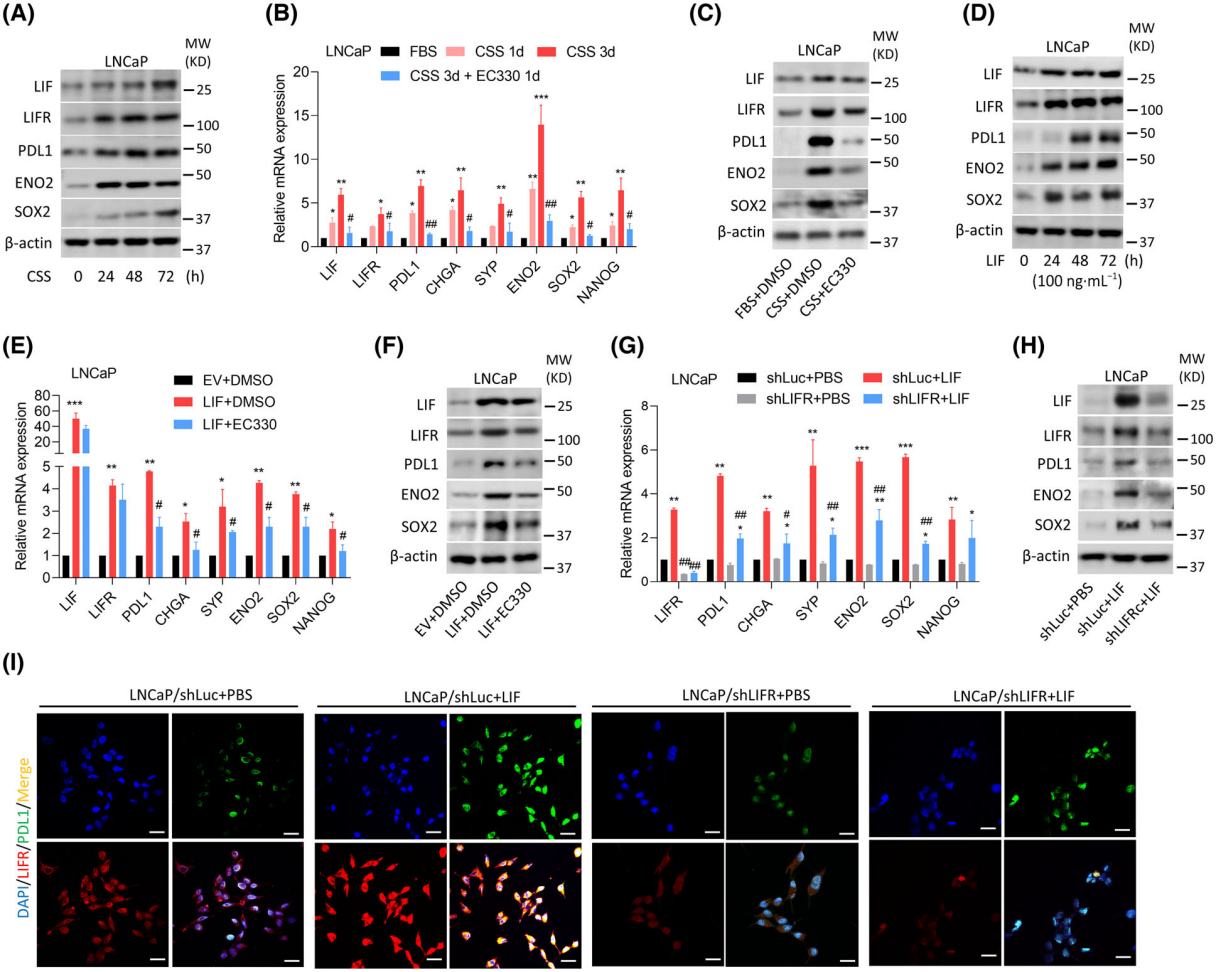
The leukemia‐inhibitory factor (LIF)/LIF receptor (LIFR)/signal transducer and activator of transcription 3 (STAT3) pathway‐driven neuroendocrine differentiation (NED) associates with programmed cell death ligand 1 (PDL1) expression in prostate cancer (PCa) after androgen deprivation therapy (ADT). (A) Protein levels of LIF, LIFR, PDL1, ENO2, and SOX2 in LNCaP cells cultured in charcoal‐stripped serum (CSS)‐containing medium were determined over 0 to 72 h from three independent experiments. (B) Relative mRNA levels of *LIF*, *LIFR*, *PDL1*, neuroendocrine (*CHGA*, *SYP*, and *ENO2*), and stem cell (*SOX2* and *NANOG*) markers in LNCaP cells cultured in CSS‐containing medium for 1 or 3 days, followed by treatment with 35 nm EC330 for 1 day. Asterisks vs. fetal bovine serum (FBS); hashtags vs. CSS for 3 days, as determined by a two‐way ANOVA and *t*‐test. (C) Protein levels of LIF, LIFR, PDL1, ENO2, and SOX2 in LNCaP cells treated with CSS‐containing medium, followed by treatment with DMSO or 35 nm EC330 for 24 h. Data determined from three independent experiments. (D) Protein levels of LIF, LIFR, PDL1, ENO2, and SOX2 in LNCaP cells treated with 100 ng·mL^−1^ of the LIF recombinant protein for 0~72 h. Data determined from three independent experiments. (E) Relative mRNA levels of *LIF*, *LIFR*, *PDL1*, *CHGA*, *SYP*, *ENO2*, *SOX2*, and *NANOG* in LNCaP cells stably expressing an empty vector (EV) or *LIF* cDNA vector, followed by treatment with DMSO or 35 nm EC330 for 24 h. Asterisks vs. EV + DMSO; hashtags vs. LIF + DMSO, as determined by a two‐way ANOVA and *t*‐test. (F) Protein levels of LIF, LIFR, PDL1, ENO2, and SOX2 in LNCaP cells expressing an EV or LIF cDNA vector, followed by treatment with DMSO or 35 nm EC330 for 24 h. Data determined from three independent experiments. (G) Relative mRNA levels of *LIFR*, *PDL1*, *CHGA*, *SYP*, *ENO2*, *SOX2*, and *NANOG* in LNCaP cells stably transfected with a non‐target control (Luc) or LIFR shRNA vector, followed by treatment with phosphate‐buffered saline (PBS) or 100 ng·mL^−1^ of the LIF recombinant protein for 48 h. Asterisks vs. PBS; hashtags vs. shLuc, as determined by a two‐way ANOVA and *t*‐test. (H) Protein levels of LIF, LIFR, PDL1, ENO2, and SOX2 in LNCaP cells stably transfected with Luc or *LIFR* shRNA, followed by treatment with PBS or 100 ng·mL^−1^ of the LIF recombinant protein for 48 h. Data determined from three independent experiments. (I) Immunofluorescence (IF) staining of LIFR and PDL1 was performed in LNCaP cells expressing Luc or LIFR shRNA vector and treated with PBS or 100 ng·mL^−1^ of LIF recombinant protein for 48 h. Antibodies specific to LIFR (red) and PDL1 (green) were employed. Nuclei were visualized with DAPI staining (blue). White scale bars represent 20 μm. Data determined from three independent experiments. Quantification of relative mRNA levels is presented as the mean ± SEM from three biological replicates. *P < 0.05, **P < 0.01, ***P < 0.005, #*P* < 0.05, ##*P* < 0.01.

Our previous results showed that activation of LIF/LIFR signaling through ADT can trigger PCa cells to undergo NED progression [16]. We further tested how ADT‐induced LIF/LIFR signaling regulates the immunosuppressive response. AR‐positive LNCaP cells were treated with CSS‐containing medium to mimic ADT, and we found that an increase in LIF/LIFR was time‐dependently associated with immune checkpoint (PDL1), neuroendocrine marker (enolase 2 (ENO2)), and stem cell marker (SRY‐box 2 (SOX2)) abundances in cells after CSS‐containing medium treatment compared to the treatment naïve group (Fig. 1A and Fig. S1A). To evaluate the effect of ADT‐induced the association of PDL1 with LIF/LIFR‐driven NED, LNCaP cells treated with CSS‐containing medium were further treated with EC330, a LIF inhibitor. Results demonstrated a correlation between the induction of PDL1, neuroendocrine, and stem cell markers and the expression of LIF and LIFR in cells treated with ADT compared to the treatment naïve group, while the LIF inhibitor reduced the abundance of these markers (Fig. 1B,C and Fig. S1B). Next, the LNCaP cells were treated with LIF protein. We observed a time‐dependent increase in the levels of LIF protein, which was associated with elevated protein levels of PD‐L1, neuroendocrine, and stem cell markers, compared to those in the untreated cells (Fig. 1D and Fig. S1C). Consistently, *LIF* overexpression in LNCaP cells produced upregulation of messenger (m)RNA and protein levels of PDL1, neuroendocrine, and stem cell markers compared to EV‐expressing cells (Fig. 1E,F and Fig. S1D). In contrast, *LIF*‐overexpressing cells exhibited significantly decreased mRNA and protein levels of these markers in response to the LIF inhibitor (Fig. 1E,F and Fig. S1D). Moreover, LNCaP cells expressing *LIFR*‐KD reduced these markers compared to the control vector (shLuc)‐expressing cells, regardless of LIF treatment (Fig. 1G,H and Fig. S1E). *LIFR*‐KD cells were subjected to LIF treatment, and LIFR and PDL1 expressions were examined through IF staining. Results demonstrated colocalization between LIFR and PDL1 in LIF‐treated cells compared to control cells. However, the effects were abolished when LIFR‐KD was applied (Fig. 1I). These results suggest that LIF/LIFR‐driven NED may be associated with immunosuppressive responses of PCa after ADT, by which LIF inhibition might suppress those effects.

### 3.2 Activation of LIF/LIFR signaling in prostate stromal cells is involved in the NED and immunosuppressive responses of PCa cells

**Fig. 2:**
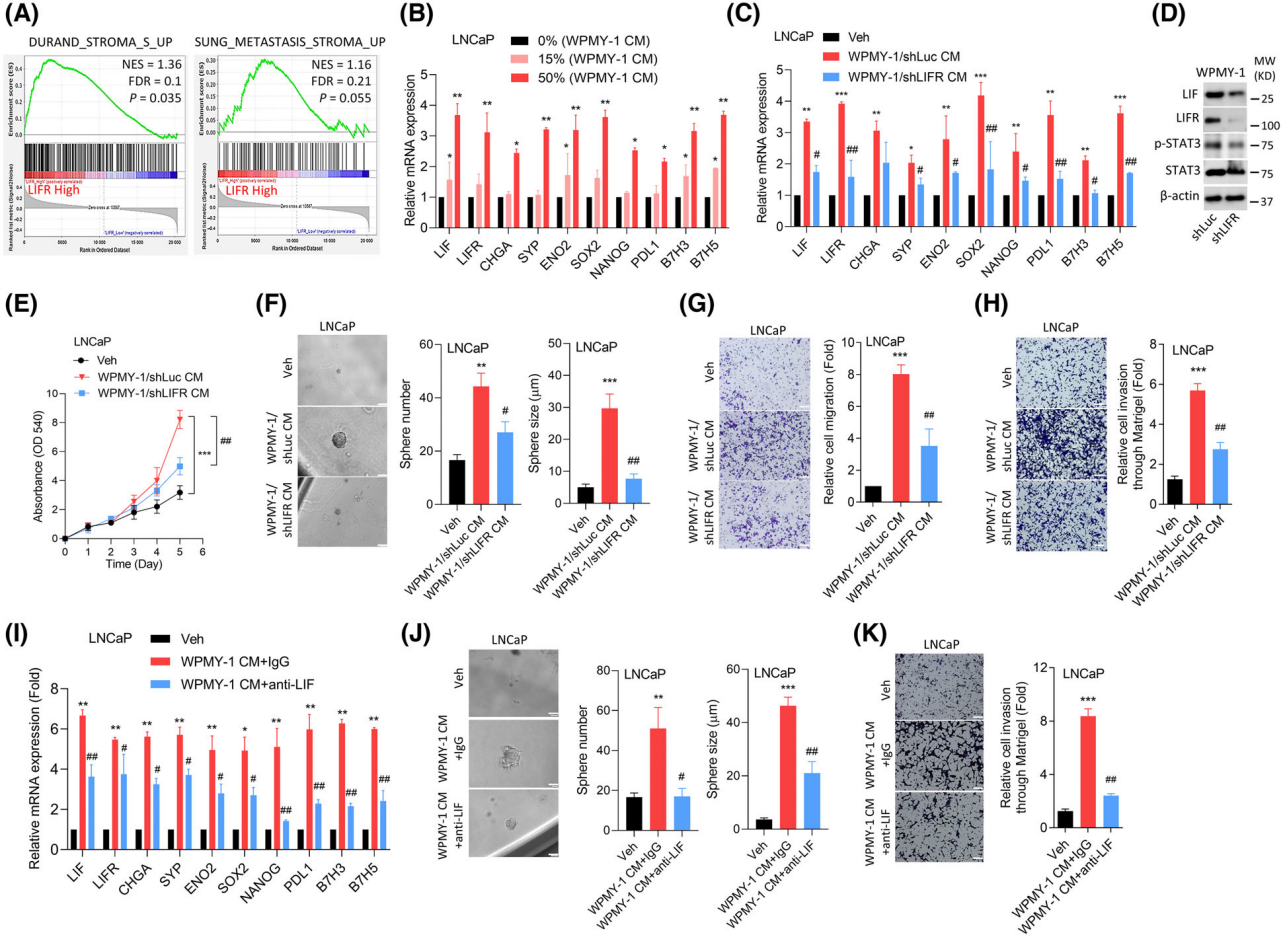
The abundance of leukemia‐inhibitory factor receptor (*LIFR*) in prostate stromal cells correlates with immune checkpoint expression and cell invasiveness in prostate cancer (PCa) cells. (A) Gene set enrichment analysis (gsea) of TCGA PCa dataset shows that high abundances of LIFR mRNAs in PCa samples were positively associated with stromal cell‐responsiveness gene signatures (Durand and Sung). FDR, false discovery rate; NES, normalized enrichment score. (B) Relative mRNA expression levels of *LIF*, *LIFR*, neuroendocrine markers (*CHGA*, *SYP*, and *ENO2*), stem cell markers (*SOX2* and *NANOG*), and immune checkpoints (*PDL1*, *B7H3*, and *B7H5*) in LNCaP cells cultured with 0%, 15%, or 50% of conditioned medium (CM) collected from human WPMY‐1 cells for 48 h. Asterisks vs. 0%, as determined by a one‐way ANOVA and *t*‐test. (C) Relative mRNA expression levels of *LIF*, *LIFR*, *CHGA*, *SYP*, *ENO2*, *SOX2*, *NANOG*, *PDL1*, *B7H3*, and *B7H5* in LNCaP cells cultured with 50% CM collected from the non‐target control (Luc) or *LIFR* shRNA‐expressing WPMY‐1 cells for 48 h. Asterisks vs. the Veh; hashtags vs. WPMY‐1/shLuc CM, as determined by a two‐way ANOVA and *t*‐test. (D) Protein levels of LIF, LIFR, p‐STAT3, and STAT3 in WPMY‐1 cells stably transfected with Luc or LIFR shRNA. Data determined from three independent experiments. (E) Cell proliferation assays of LNCaP cells cultured with 50% CM collected from the Luc or *LIFR* shRNA‐expressing WPMY‐1 cells for 5 days. *n* = 8 per group. Asterisks vs. Veh; hashtags vs. WPMY‐1/shLuc CM, by a two‐way ANOVA. (F) Relative number and size of sphere formation was assessed in LNCaP cells cultured with CM collected from Luc or *LIFR* shRNA‐expressing WPMY‐1 cells for 1 week. Scale bars represent 20 μm. Asterisks vs. Veh; hashtags vs. WPMY‐1/shLuc CM, as determined by a two‐way ANOVA and *t*‐test. (G, H) Relative cell migration (G) and invasion through Matrigel (H) were assessed in LNCaP cells cultured with CM collected from Luc or *LIFR* shRNA‐expressing WPMY‐1 cells for 12 h. Scale bars represent 100 μm. Asterisks vs. Veh; hashtags vs. WPMY‐1/shLuc CM, by a two‐way ANOVA. (I) Relative mRNA expression levels of *LIF*, *LIFR*, *CHGA*, *SYP*, *ENO2*, *SOX2*, *NANOG*, *PDL1*, *B7H3*, and *B7H5* in LNCaP cells cultured with 50% CM collected from WPMY‐1 cells and treated with 1 μg·mL^−1^ of an LIF antibody or control IgG for 48 h. * vs. Veh; hashtags vs. WPMY‐1 CM + IgG, as determined by a two‐way ANOVA and *t*‐test. (J, K) Relative number and size of sphere formation (J) and invasion through Matrigel (K) were assessed in LNCaP cells cultured with CM collected from WPMY‐1 cells and treated with 1 μg·mL^−1^ of an LIF antibody or control IgG for 1 week (J) and 12 h (K), respectively. Scale bars represent 20 μm. Asterisks vs. the Veh; hashtags vs. WPMY‐1 CM + IgG, by a two‐way ANOVA. Quantification of relative mRNA levels, migration, invasion, proliferation, and sphere formation data are presented as the mean ± SEM from three biological replicates. *P < 0.05, **P < 0.01, ***P < 0.005, #*P* < 0.05, ##*P* < 0.01.

Prostate stromal cells are vital in promoting the development and proper operation of the prostate gland [23]. The dynamic and intricate TME formed by the interplay between these stromal cells and cancer cells can significantly impact tumor advancement, the development of resistance to therapies, and the ability to evade immune reactions [34]. To explore the influence of the interaction between prostate stromal cells and the abundance of LIFR in PCa cells, we investigated potential associations between elevated LIFR expression in PCa tissues and gene signatures indicative of stromal cells. This investigation was conducted using the gsea on TCGA PCa dataset. Results from the gsea revealed positive connections between high LIFR levels and upregulation of gene signatures related to stromal cells (Fig. 2A). The experiments were performed using CM collected from WPMY‐1 cells, a human prostate stromal cell line, and cultured with LNCaP cells. LNCaP cells cultured with WPMY‐1 CM revealed a significant increase in LIF/LIFR expression, which was associated with elevated mRNA levels of neuroendocrine markers (chromogranin A (*CHGA*), synaptophysin (*SYP*), and *ENO2*), stem cell markers (*SOX2* and Nanog homeobox (*NANOG*)), and immune checkpoints (*PDL1*, B7 homolog 3 (*B7H3*), and *B7H5*) (Fig. 2B). Moreover, CM collected from WPMY‐1 cells expressing *LIFR*‐KD exhibited significant reduction in these markers in PCa cells (Fig. 2C), indicating the role of LIF/LIFR signaling in human prostate stromal cells in promoting neuroendocrine and immunosuppressive marker abundance of PCa cells. The protein levels of LIF, LIFR, phosphorylated (p)‐STAT3, and STAT3 were measured in WPMY‐1 cells expressing *LIFR*‐KD or control shRNA (Fig. 2D and Fig. S1F). Additionally, CM obtained from WPMY‐1 cells expressing non‐target control (Luc) shRNA stimulated cell proliferation and the formation of cell spheres in LNCaP cells compared to control cells (Fig. 2E,F). Conversely, diminished cell proliferation and reduced sphere formation were observed in LNCaP cells when cultured with CM from WPMY‐1 cells expressing *LIFR*‐KD (Fig. 2E,F). Furthermore, when LNCaP cells were cultured with CM collected from WPMY‐1 cells expressing *LIFR*‐KD, there was a significant decrease in cell migration and invasion through Matrigel (Fig. 2G,H). Additionally, substantial decreases in the expression of these markers, as well as in sphere formation and cell invasion through Matrigel, were observed in LNCaP cells cultured in CM collected from WPMY‐1 cells treated with an anti‐LIF antibody, compared to control cells (Fig. 2I–K). These results suggest that cytokines secreted by prostate stromal cells might induce the expression of neuroendocrine and immunosuppressive markers, which are linked to the aggressive progression of PCa cells.

We next examined the association between LIFR and immunosuppressive‐responsive gene signatures using a GSEA of TCGA PCa dataset. Immunosuppressive cytokines (such as IL‐10 and TGF‐ß) were shown to suppress antitumor immune responses that contribute to immune evasion by cancer cells and facilitate tumor progression [35, 36]. We found that tissues expressing high levels of the LIFR were positively associated with gene signatures of IL‐10 and TGF‐ß responsiveness (Fig. S2A). A correlation analysis showed that LIFR expression was positively associated with PDL1 expression in TCGA PCa datasets (Fig. S2B). We further investigated the association between immune infiltration and LIFR expression in the immune microenvironment using a prostate adenocarcinoma (PRAD) dataset of the TIMER database [37]. We found that LIFR‐abundant PCa cells were positively associated with infiltration levels of immunosuppressive immune cell types in the PCa TME, such as CAFs, M2 macrophages, and Treg cells (Fig. S2C). However, proinflammatory immune cell types, such as M1 macrophages, cluster of differentiation 8‐positive (CD8^+^)/CD4^+^ T‐cells, dendritic cells, NK cells, and NK T‐cells were inversely correlated with LIFR expression (Fig. S2D). These findings suggest that activation of the LIF/LIFR axis contributes to NED and malignant progression, potentially resulting in the upregulation of immunosuppressive responses in the PCa TME.

### 3.3 ADT‐driven LIF/LIFR signaling induces the BDNF expression

**Fig. 3:**
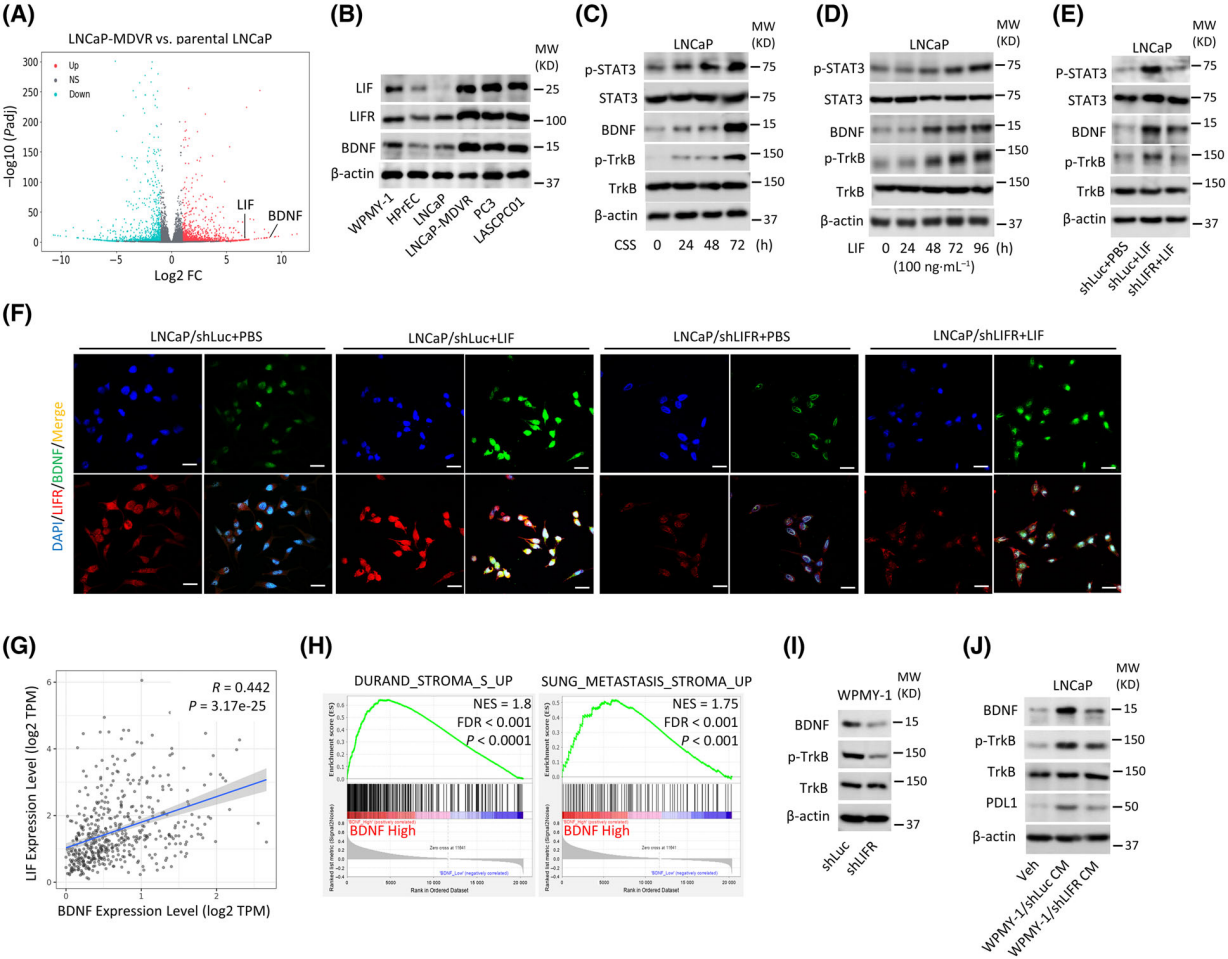
Androgen‐deprivation therapy (ADT) promotes the activation of leukemia‐inhibitory factor (*LIF*)/LIF receptor (*LIFR*)/signal transducer and activator of transcription 3 (STAT3) signaling, which is linked to the induction of BDNF in prostate cancer (PCa) cells. (A) Volcano plot analysis of expressed genes was conducted using RNA‐sequencing data from a set of samples collected from MDV3100‐resistant LNCaP‐MDVR cells compared to parental LNCaP cells. The *BDNF* and *LIF* genes were significantly upregulated in a population of positively enriched LNCaP‐MDVR cells. (B) Protein levels of LIF, LIFR, and BDNF in prostate stromal cells (WPMY‐1), normal prostate epithelial cells (HPrEC), prostate adenocarcinoma cells (LNCaP and PC3), MDV3100‐resistant cells (LNCaP‐MDVR), and NEPC‐like (LASCPC01) cells. Data determined from three independent experiments. (C) Protein levels of p‐STAT3, STAT3, BDNF, p‐TrkB, and TrkB in LNCaP cells cultured in charcoal‐stripped serum (CSS)‐containing medium for 0~72 h. Data determined from three independent experiments. (D) Protein levels of p‐STAT3, STAT3, BDNF, p‐TrkB, and TrkB in LNCaP cells treated with 100 ng·mL^−1^ of the LIF recombinant protein for 0~96 h. (E) Protein levels of p‐STAT3, STAT3, BDNF, p‐TrkB, and TrkB in LNCaP cells stably transfected with a non‐target control (Luc) or *LIFR* shRNA vector, followed by treatment with phosphate‐buffered saline (PBS) or 100 ng·mL^−1^ of the LIF recombinant protein for 48 h. Data determined from three independent experiments. (F) Immunofluorescence (IF) staining was performed to assess abundances of LIFR and BDNF in parental LNCaP cells or LNCaP cells expressing *LIFR*‐KD, followed by treatment with PBS or 100 ng·mL^−1^ of the LIF recombinant protein for 48 h. Antibodies specific to LIFR (red) and BDNF (green) were employed. Nuclei were visualized using DAPI staining (blue). White scale bars represent 20 μm. Data determined from three independent experiments. (G) Correlation analyses of LIF and BDNF mRNA levels in PCa tissue samples from TCGA PCa datasets. Correlation coefficients (*R* ^2^) and *P*‐values were determined by correlation XY analyses in graphpad prism. *R* = 0.442; *P* = 3.17e‐25. (H) Gene Set Enrichment Analysis (gsea) of TCGA PCa dataset showing that high abundances of BDNF mRNAs in PCa samples were positively associated with stroma cell (Durand and Sung) responsiveness gene signatures. FDR, false discovery rate; NES, normalized enrichment score. (I) Protein levels of BDNF, p‐TrkB, and TrkB in WPMY‐1 cells stably transfected with Luc or LIFR shRNA. (J) Protein levels of BDNF, p‐TrkB, TrkB, and PDL1 in LNCaP cells cultured with conditioned medium (CM) collected from Luc or LIFR shRNA‐expressing WPMY‐1 cells for 48 h. Data determined from three independent experiments.

ADT was shown to upregulate immune checkpoint molecules on PCa cells and the immunosuppressive response within the TME [38]. ADT can also alter the stromal and extracellular matrix components of the PCa TME, create a physical barrier, and impede immune cell infiltration into the tumor [39, 40]. High BDNF levels were associated with an advanced tumor stage, increased aggressiveness, and poor prognoses of PCa patients [21]. However, the relationship between BDNF abundance and immunosuppression in the ADT‐resistant PCa TME remains unclear. Next, we performed an RNA‐Seq analysis to identify the differentially expressed genes (DEGs) between ADT‐resistant cells and parental cells. Our analysis demonstrated a significant upregulation of both LIF and BDNF in MDV3100‐resistant LNCaP‐MDVR cells (Fig. 3A). We next measured protein expression levels of BDNF, LIF, and LIFR in prostate stromal cells and various PCa cell lines. In addition to observing high levels of BDNF associated with LIF and LIFR in WPMY‐1 cells, we also noted that increased BDNF expression was linked to higher levels of LIF and LIFR in MDV3100‐resistent LNCaP‐MDVR cells, androgen‐independent PC3 cells, and NEPC‐like LASCPC01 cells compared to the normal prostate epithelial cell HPrEC (Fig. 3B and Fig. S3A), indicating a potential association between BDNF induction and activation of LIF/LIFR signaling in advanced PCa TME. After demonstrating the induction of LIF/LIFR by ADT (Fig. 1A), we proceeded to assess the protein expression levels of p‐STAT3, STAT3, BDNF, p‐TrkB, and TrkB in androgen‐dependent LNCaP cells treated with CSS‐containing medium. Our findings revealed an increase in p‐STAT3, accompanied by elevated expression of BDNF and p‐TrkB in LNCaP cells subjected to CSS‐containing medium (Fig. 3C and Fig. S3B). To investigate the role of BDNF in the activation of LIF/LIFR signaling, LNCaP cells were exposed to LIF protein. This treatment resulted in increased levels of p‐STAT3, correlating with elevated BDNF and p‐TrkB (Fig. 3D and Fig. S3C). However, CSS or LIF‐treated cells combined with LIF inhibitor treatment abolished p‐STAT3 and was associated with decreased BDNF and p‐TrkB (Fig. S3D–G). This indicates that LIF/LIFR/STAT3 signaling may play a regulatory role in the activation of BDNF in PCa cells following ADT. In addition, when LIFR‐KD was introduced in LIF‐treated cells, there was a notable decrease in the expression levels of p‐STAT3, BDNF, and p‐TrkB (Fig. 3E and Fig. S3H). Next, we performed IF staining to assess the expression and localization of LIFR and BDNF in LNCaP cells. A significant induction of LIFR and BDNF staining intensities was found in cells treated with LIF (Fig. 3F). However, in LNCaP cells expressing *LIFR*‐KD, we observed a significant reduction in LIFR and BDNF staining intensities, regardless of LIF treatment (Fig. 3F), suggesting that BDNF induction was dependent on LIF/LIFR signaling. We examined the association between BDNF and LIF‐responsive gene signatures using a gsea of TCGA PCa dataset. We found that tissues expressing high BDNF levels were positively associated with LIF‐responsive gene signatures (Fig. S3I). Interestingly, correlation analyses in TCGA PCa dataset revealed a positive association between *LIF* and *BDNF* mRNA levels (Fig. 3G), suggesting that BDNF abundance may be regulated by LIF signaling. Furthermore, BDNF expression in PCa cells was positively associated with upregulation of gene signatures related to stromal cells (Fig. 3H), as revealed by a gsea in patients with high BDNF levels in the TCGA PCa dataset. These findings highlight the potential role of LIF/LIFR signaling in mediating the activation of BDNF and its contribution to the development of ADT resistance in PCa TME.

To examine the role of the LIF pathway in promoting an immunosuppressive phenotype through the induction of BDNF in prostate stromal cells, we utilized WPMY‐1 cells expressing control or LIFR‐KD. Results showed that the protein amounts of BDNF and p‐TrkB are reduced in WPMY‐1 cells expressing LIFR‐KD compared with control cells (Fig. 3I and Fig. S3J). Cell culture supernatants were collected from WPMY‐1 cells expressing control or LIFR‐KD to be treated with LNCaP cells. When exposed to conditioned medium from control WPMY‐1 cells, showed a greater induction of BDNF, p‐TrkB, and PDL1 protein expression compared to the vehicle control group (Fig. 3J and Fig. S3K). Conversely, treatment with conditioned medium from WPMY‐1 cells expressing *LIFR*‐KD resulted in a decrease in the expression of BDNF, p‐TrkB, and PDL1 proteins compared to the group of WPMY‐1 cells expressing the control vector (Fig. 3J and Fig. S3K). Our results suggest that the LIF/LIFR/STAT3 pathway plays a pivotal role in promoting an immunosuppressive phenotype in both PCa cells and prostate stromal cells through the induction of BDNF in the TME. In response to ADT‐triggered activation of the LIF/LIFR/SATA3 pathway, BDNF may be secreted by both PCa cells and prostate stromal cells within the TME.

### 3.4 BDNF drives the NED and aggressiveness of PCa cells

Our previous report has demonstrated the upregulation of neuroendocrine marker expression in androgen‐dependent LNCaP cells treated with long‐term MDV3100 [41, 42]. Our findings are consistent with other studies that used the same cells under similar culture conditions [43]. To test the concept that BDNF contributes to immunosuppressive and malignant phenotypes in MDV3100‐resistant LNCaP cells, we subsequently knocked down BDNF expression in LNCaP‐MDVR cells. The results demonstrated that *BDNF*‐KD expression in LNCaP‐MDVR cells leads to a suppression of neuroendocrine, stem cell, and immune checkpoint markers (Fig. S4A). Additionally, BDNF‐KD expression in the same cells results in reduced cell proliferation, migration, and invasion compared to cells expressing the control vector (Fig. S4B–D). Our research demonstrated a notable association between the increased expression of BDNF and the malignant progression observed in MDV3100‐resistant PCa cells.

**Fig. 4:**
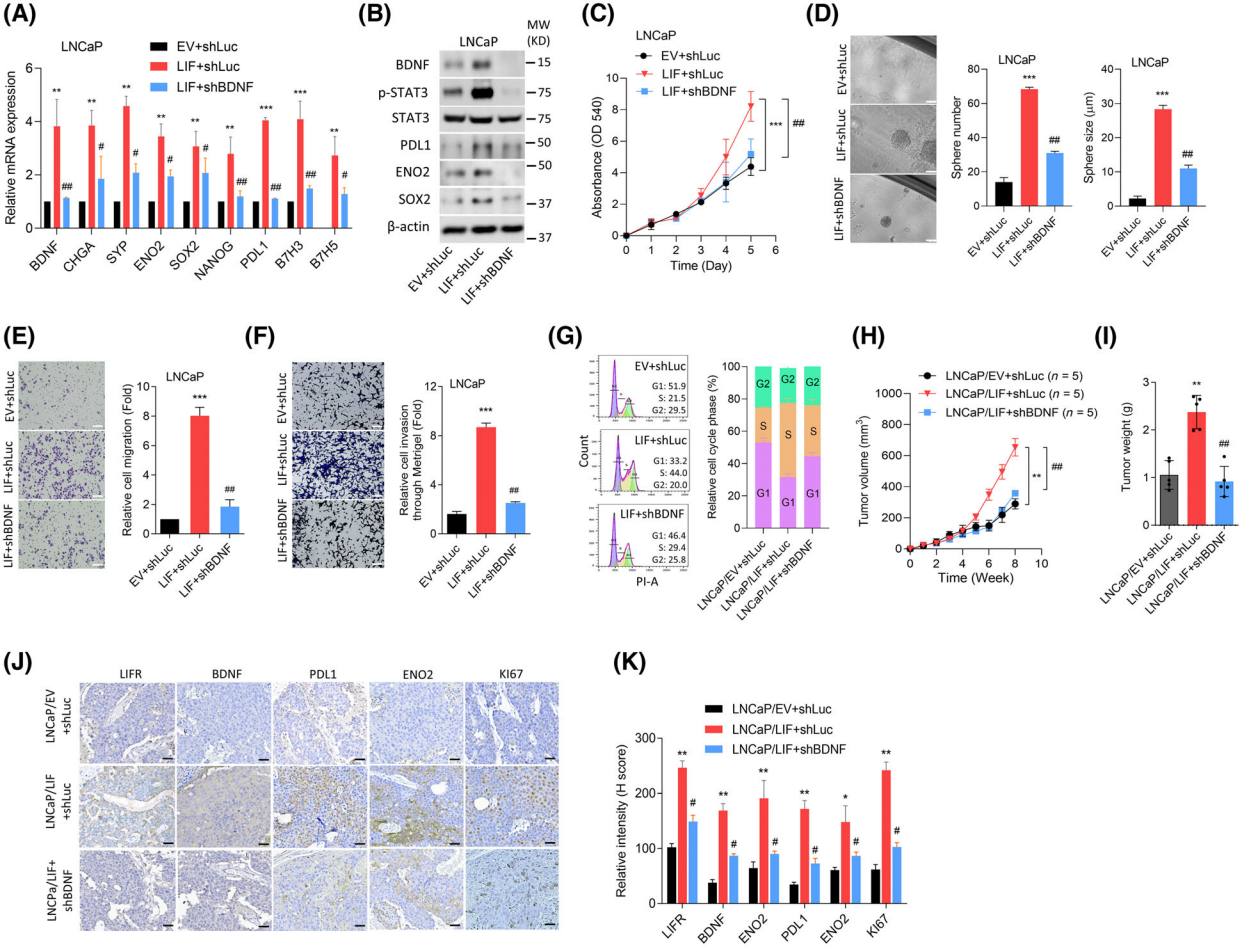
The abundance of brain‐derived neurotrophic factor (*BDNF*) is regulated by leukemia‐inhibitory factor (*LIF*) and is associated with the neuroendocrine differentiation (NED) and aggressiveness of prostate cancer (PCa) cells. (A) Relative mRNA levels of *BDNF*, neuroendocrine (*CHGA*, *SYP*, and *ENO2*), stem cell (*SOX2* and *NANOG*), and immune checkpoint (*PDL1*, *B7H3*, and *B7H5*) markers in LNCaP cells stably transfected with an empty vector (EV) or LIF cDNA vector, followed by stable transfection with a non‐target control (Luc) vector or BDNF shRNA vector. Asterisks vs. EV + shLuc; hashtags vs. LIF + shLuc, as determined by a two‐way ANOVA and *t*‐test. (B) Protein levels of BDNF, p‐STAT3, STAT3, PDL1, ENO2, and SOX2 in LNCaP cells stably transfected with the EV or LIF cDNA vector, followed by stable transfection with Luc or BDNF shRNA vector. Data determined from three independent experiments. (C, D) Cell proliferation assays (C) and relative number and size of sphere formation (D) of LNCaP cells stably transfected with the EV or LIF cDNA vector, followed by stable transfection with Luc or BDNF shRNA vector for 5 days (C) and 1 week (D), respectively. *n* = 8 per group. Asterisks vs. EV + shLuc; hashtags vs. LIF + shLuc, as determined by a two‐way ANOVA and *t*‐test. Scale bars represent 20 μm. (E, F) Relative cell migration (E) and invasion through Matrigel (F) were assessed in LNCaP cells stably transfected with an EV or *LIF* cDNA vector, followed by stable transfection with Luc or BDNF shRNA vector for 12 h. Scale bars represent 100 μm. Asterisks vs. EV + shLuc; hashtags vs. LIF + shLuc, as determined by a two‐way ANOVA and *t*‐test. (G) Cell cycle analysis by flow cytometry was assessed in LNCaP cells stably transfected with an EV or *LIF* cDNA vector, followed by stable transfection with Luc or BDNF shRNA vector. *n* = 3 per group. (H, I) Tumor growth analysis was conducted on LNCaP cells expressing EV + shLuc, LIF + shLuc, and LIF + shBDNF by subcutaneously inoculating cells into male nude mice, allowing them to grow for 8 weeks. Tumor sizes were measured weekly (H). Tumor weights were measured upon tumor collection (I). *n* = 5 per group. Asterisks vs. the EV + shLuc; hashtags vs. LIF + shLuc, as determined by a one‐way ANOVA and *t*‐test. (J, K) Immunohistochemical (IHC) staining and intensity analyses were performed to assess protein levels of LIFR, BDNF, PDL1, ENO2, and KI67 in subcutaneous tumors derived from (I). Asterisks vs. LNCaP/EV + shLuc; hashtags vs. LNCaP/LIF + shLuc. Intensities were compared using a two‐tailed Student's *t*‐test. Scale bars, 100 μm. Quantification of relative mRNA levels, migration, invasion, and proliferation data are presented as the mean ± SEM from three biological replicates. *P < 0.05, **P < 0.01, ***P < 0.005, #*P* < 0.05, and ##*P* < 0.01.

We aimed to clarify the molecular mechanisms governing the regulation of BDNF expression through the LIF/LIFR/STAT3 pathway and its potential involvement in immune evasion of PCa TME. We found that upregulation of *BDNF* mRNA was associated with increased expression of neuroendocrine markers (*CHGA*, *SYP*, and *ENO2*), stemness markers (*SOX2* and *NANOG*), and immune checkpoint markers (*PDL1*, *B7H3*, and *B7H5*) in LNCaP cells overexpressing *LIF* cDNA (Fig. 4A). Interestingly, significant downregulation of these markers was observed in LNCaP cells expressing *BDNF*‐KD, regardless of LIF overexpression (Fig. 4A). A western blot analysis confirmed that higher BDNF protein expression was positively associated with the p‐STAT3, PDL1, ENO2, and SOX2 proteins in LNCaP cells overexpressing LIF; however, BDNF‐KD partially reduced the effects of LIF, resulting in reduced expression of those proteins (Fig. 4B and Fig. S4E). Cell proliferation and sphere formation assays demonstrated an increase in the growth rate of LNCaP cells expressing LIF compared to control cells; however, BDNF‐KD reduced LIF‐driven cell growth (Fig. 4C,D). Cell migration and invasion through Matrigel assays revealed enhanced cell motility in LNCaP cells with LIF overexpression (Fig. 4E,F). In contrast, these effects were significantly diminished in cells with BDNF‐KD compared to control cells (Fig. 4E,F). Interestingly, overexpression of LIF resulted in a reduction in the G1 phase and an increase in the S phase, as validated by cell cycle analyses (Fig. 4G). However, these effects were reduced when LIF‐overexpressed cells underwent *BDNF*‐KD (Fig. 4G). These observations imply that the activation of the LIF/BDNF signaling may contribute to the regulation of cell proliferation, migration, invasion, and division. Xenograft mouse models were established by injecting LNCaP cells expressing control, *LIF* cDNA vector, or *LIF*‐overexpressing cells with *BDNF*‐KD. A tumor growth analysis revealed accelerated tumor growth in mice injected with LIF‐overexpressing cells compared to control cells; however, a reduction in the tumor growth rate and tumor weight were found in mice injected with cells expressing *BDNF*‐KD (Fig. 4H,I). IHC analysis of tumor sections demonstrated increased expression of LIFR, BDNF, PDL1, ENO2, and KI67 in LIF‐overexpressing tumors compared to control tumors; however, BDNF‐KD eliminated the LIF‐driven expression intensity (Fig. 4J,K and Fig. S4F). Our results demonstrated that BDNF activation contributes to enhanced proliferation, migration, invasion, and tumor growth of PCa cells and might be associated with NED and immune checkpoints abundance.

### 3.5 Regulation of *BDNF* and *PDL1* by LIF/LIFR/STAT3 signaling in PCa

**Fig. 5:**
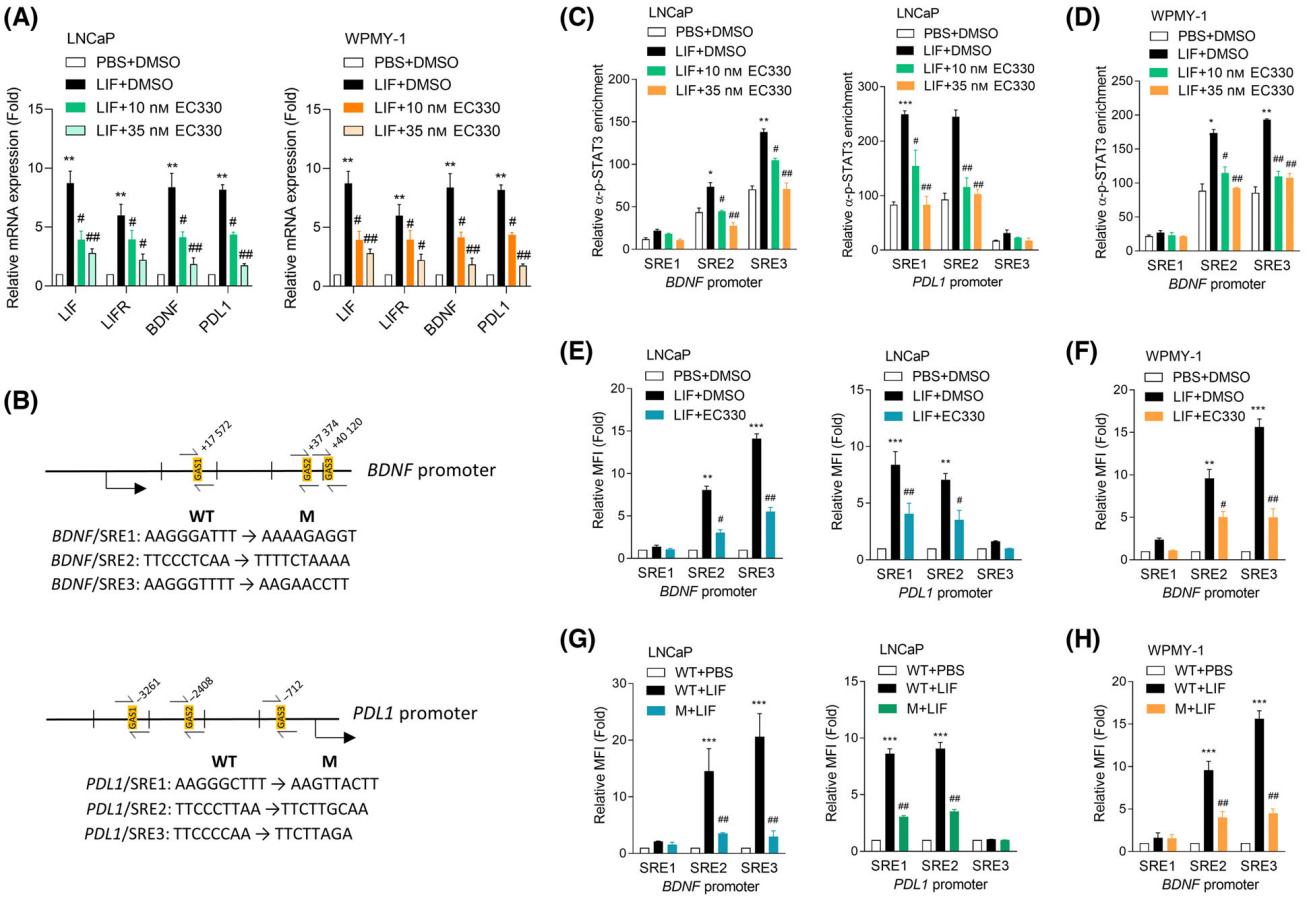
Brain‐derived neurotrophic factor (*BDNF*) and programmed death‐ligand 1 (*PDL1*) are regulated by leukemia‐inhibitory factor (LIF)/LIF receptor (LIFR)/signal transducer and activator of transcription 3 (STAT3) signaling in prostate cancer (PCa) and prostate stromal cells. (A) Relative mRNA levels of LIF, LIFR, BDNF, and PDL1 in LNCaP and WPMY‐1 cells treated with phosphate‐buffered saline (PBS) or 100 ng·mL^−1^ of the LIF protein or combined with DMSO or 10 or 35 nm EC330 treatment for 48 h. Asterisks vs. PBS + DMSO; hashtags vs. LIF + DMSO, as determined by a two‐way ANOVA and *t*‐test. (B) Schematic of the predicted STAT3‐response element (SRE) and an introduced single‐binding site mutant in regulatory sequence reporter constructs of human *BDNF* (GRCh38:11) and *PDL1* (GRCh38:9). (C, D) Chromatin immunoprecipitation (ChIP) assay showing binding of p‐STAT3 to predicted SREs of the *BDNF* and *PDL1* genes regulatory sequence in LNCaP (C) or WPMY‐1 (D) cells treated with PBS or 100 ng·mL^−1^ of the LIF protein or combined with DMSO or 10 or 35 nm EC330 treatment for 24 h. Sheared chromatin from nuclear extracts was precipitated with antibodies to p‐STAT3 and predictive primers (B, black arrows) were used to quantify the precipitated DNA by a qPCR. Enrichment of each protein to each site is given as a percentage of the total input and then normalized to IgG. Asterisks vs. PBS + DMSO; hashtags vs. LIF + DMSO, as determined by a two‐way ANOVA and *t*‐test. (E, F) The relative mean fluorescence intensity (MFI) of the green fluorescent protein (GFP) reporter gene containing wild‐type (WT)‐SREs from the *BDNF* or *PDL1* regulatory sequence in LNCaP (E) or WPMY‐1 (F) cells treated with PBS or 100 ng·mL^−1^ of the LIF protein or combined with DMSO or 35 nm EC330 treatment for 48 h. Asterisks vs. PBS + DMSO; hashtags vs. LIF + DMSO, as determined by a two‐way ANOVA and *t*‐test. (G, H) Relative MFI of the GFP reporter gene containing WT‐ or mutant (M)‐SREs from the *BDNF* or *PDL1* regulatory sequence in LNCaP (G) or WPMY‐1 (H) cells treated with PBS or 100 ng·mL^−1^ of the LIF protein for 48 h. Asterisks vs. WT + PBS; hashtags vs. WT + LIF, as determined by a two‐way ANOVA and *t*‐test. Quantification of the ChIP assay and relative MFI values are presented as the mean ± SEM from three biological replicates. *P < 0.05, **P < 0.01, ***P < 0.005, #*P* < 0.05, and ##*P* < 0.01.

To investigate the roles of LIF/LIFR‐driven *BDNF* and *PDL1* transcription, we examined mRNA levels of *LIF*, *LIFR*, *BDNF*, and *PDL1* in LNCaP cells treated with the LIF protein and subsequently treated with an LIF inhibitor EC330. Results demonstrated significant increases in *LIF*, *LIFR*, *BDNF*, and *PDL1* mRNA levels in cells treated with the LIF protein; however, these effects were abolished when EC330 was used (Fig. 5A, left). Intriguingly, we also observed elevated mRNA expression of *LIF*, *LIFR*, and *BDNF* in WPMY‐1 cells following LIF treatment, and this effect was reversed when a LIF inhibitor was applied (Fig. 5A, right). These findings suggest that a comparable regulatory mechanism may exist in both PCa and prostate stromal cells, whereby LIF‐driven signaling may enhance *BDNF* and *PDL1* transcription. To study the regulation of *BDNF* and *PDL1* by LIF/LIFR/STAT3 signaling, we validated the direct binding of STAT3 to promoters of *BDNF* and *PDL1*. ChIP‐sequencing data were downloaded from the GEO (GSM2752894 and GSM2752900) and analyzed by Genomic blower (UCSC) to identify genome‐wide binding sites of STAT3. The analysis revealed significant enrichment of STAT3 binding at genomic loci corresponding to *BDNF* and *PDL1* (Fig. S5A,B), suggesting potential direct regulation of these genes by STAT3. We next searched for sequences resembling the STAT3 response element (SRE) in *BDNF* and *PDL1* regulatory sequences through the PROMO database of transcription factor‐binding profiles [44]. We found three putative SREs downstream relative to the *BDNF* transcription start site and three SREs upstream relative to the *PDL1* transcription start site (Fig. 5B). ChIP assays were performed using antibodies specific for p‐STAT3 or IgG in LNCaP cells treated with EC330 following LIF protein treatment. Results revealed a significant enrichment of p‐STAT3 binding to SRE2 and SRE3 of *BDNF* and SRE1 and SRE2 of *PDL1* in cells with LIF treatment, compared to control IgG (Fig. 5C). However, the binding ability of p‐STAT3 was reduced in cells with additional EC330 treatment (Fig. 5C), confirming that *BDNF* and *PDL1* are regulated by LIF/LIFR/STAT3 signaling in PCa cells. We further clarified whether activation of LIF/LIFR/STAT3 signaling regulates BDNF and PDL1 in WPMY‐1 cells. Consistently, we found that the p‐STAT3‐binding capacities of SRE2 and SRE3 to *BDNF* significantly increased after LIF protein treatment but decreased in EC330‐treated WPMY‐1 cells (Fig. 5D). Reporter assays were performed using a DNA construct containing either wild‐type (WT) or mutant (M) SRE on *BDNF* or *PDL1* regulatory sequences cloned into a GFP reporter in LNCaP or WPMY‐1 cells. We observed significant increases in SRE2 and SRE3 activities of the *BDNF*‐GFP reporter and SRE1 and SRE2 activities of the *PDL1*‐GFP reporter in LIF protein‐treated LNCaP cells compared to untreated cells (Fig. 5E). Additionally, WPMY‐1 cells exhibited elevated SRE2 and SRE3 activities of the *BDNF*‐GFP reporter upon LIF protein treatment (Fig. 5F). Conversely, the effects of LIF in both cells were reduced when treated with EC330 (Fig. 5E,F). We also observed that introduced mutations in SRE2 and SRE3 of the *BDNF*‐GFP reporter and SRE1 and SRE2 of the *PDL1*‐GFP reporter resulted in the reduction of LIF‐driven reporter activity in LNCaP cells (Fig. 5G). Similarly, in WPMY‐1 cells, expression of the *BDNF*‐GFP reporter with SRE2 and SRE3 mutations resulted in decreased LIF‐driven reporter activity (Fig. 5H). Our findings suggest that LIF/LIFR/STAT3 signaling plays a crucial role in regulating BDNF in PCa cells and prostate stromal cells and regulating PDL1 expression in PCa cells.

### 3.6 BDNF abundance in CRPC correlates with the LIF/LIFR‐driven PDL1 expression

**Fig. 6:**
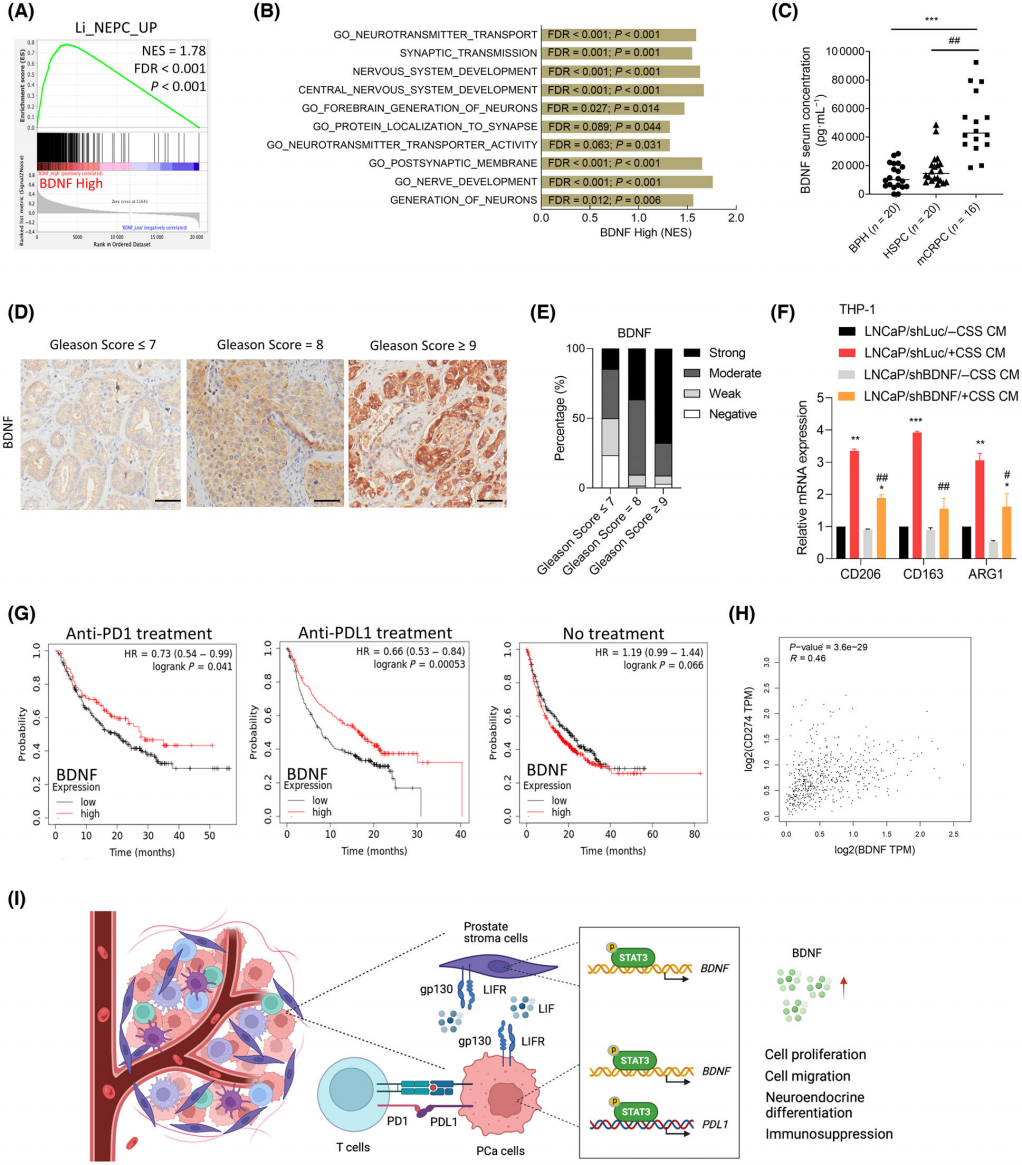
Brain‐derived neurotrophic factor (BDNF) abundance in the serum of castration‐resistant prostate cancer (CRPC) patients is correlated with neuroendocrine differentiation (NED) of prostate cancer (PCa). (A, B) Gene set enrichment analysis (gsea) of TCGA PCa dataset show that a high abundance of BDNF mRNAs in PCa samples was positively associated with gene signatures representing NEPC responsiveness (A, Li) and neuronal development (B). FDR, false discovery rate; NES, normalized enrichment score. (C) Relative BDNF concentrations in patient sera derived from benign prostatic hyperplasia (BHP; *n* = 20), hormone‐sensitive PCa (HSPC, *n* = 20), and metastatic CRPC samples (*n* = 16). Patient serum samples were collected at the Taipei Medical University‐Affiliated Hospital in Taipei. Asterisks vs. BPH; hashtags vs. HSPC, as determined by a two‐way ANOVA and *t*‐test. (D) Representative images of immunohistochemical (IHC) staining of BDNF in selected tissue sections of the PCa tissue microarray (TMA) with different Gleason scores (*n* = 40, PR483d, TissueArray.Com LLC). Scale bars, 100 μm. (E) Relative intensity of IHC staining from (D). The *P*‐values were calculated by a Chi‐squared test performed using spss statistical 18.0 software. *P* < 0.001. (F) Relative mRNA expression levels of CD206, CD163, and ARG1 in THP‐1 cells cultured with conditioned medium (CM) collected from the non‐target control (Luc) or BDNF shRNA‐expressing LNCaP cells cultured in CSS‐containing medium for 5 days. Asterisks vs. ‐CSS; hashtags vs. shLuc, as determined by a two‐way ANOVA and *t*‐test. (G) Survival analysis compared overall survival rates between patients with high‐BDNF expression and those with low‐BDNF expression, including those who may have undergone anti‐PD1 treatment (left), anti‐PDL1 treatment (middle), or no treatment (right). (H) Pearson correlations between *BDNF* and *PDL1/CD274* were analyzed in TCGA PCa dataset using XY correlation analyses in graphpad prism. (I) Schematic summary of this study. LIF/LIFR/STAT3 signaling activation is associated with the induction of BDNF in the PCa TME, which was correlated with increased PDL1 expression and NED features in PCa cells. LIF/LIFR/STAT3 activation in prostate stromal cells played a crucial role in the induction of *BDNF* and *PDL1* expression, highlighting the importance of immunosuppressive TME in regulating immune checkpoint expressions. BDNF induction may link to immunosuppression in PCa, offering potential prognostic markers and therapeutic targets to counter immune evasion and NED in advanced stages of PCa. Quantification of relative mRNA levels and BDNF concentrations is presented as the mean ± SEM from three biological replicates. **P < 0.01, ***P < 0.005, #*P* < 0.05, and ##*P* < 0.01.

Our investigation focused on understanding the correlation between BDNF abundance and its role in the NED progression of PCa. Through a GSEA of TCGA PCa database, we observed a positive association between higher expression levels of *BDNF* mRNA and upregulation of the NEPC‐responsive gene signature (Fig. 6A). Additionally, we found a positive association between highly abundant BDNF in PCa samples and gene signatures related to neuronal developmental responsiveness (Kyoto Encyclopedia of Genes and Genomes (KEGG), gene ontology (GO), and REACTOME) based on GSEA verification using TCGA PCa dataset (Fig. 6B). These findings support the hypothesis that upregulation of BDNF is linked to NEPC development. To explore correlations of BDNF abundance in liquid biopsies among PCa patients, we employed ELISA to quantify BDNF protein levels in serum samples from patients with BPH, HSPC, and metastatic CRPC. Results demonstrated a significant increase in BDNF levels in serum samples from metastatic CRPC patients compared to both BPH and HSPC samples (Fig. 6C), suggesting a connection between elevated BDNF levels and advanced disease stages. To further investigate the role of BDNF and its association with disease progression in PCa patients, we examined BDNF expression in a PCa TMA using IHC staining analyses. In high‐grade samples (Gleason score ≧ 9), BDNF expression was significantly upregulated compared to low‐grade (Gleason score ≦ 7) samples, suggesting a potential role of BDNF in PCa progression (Fig. 6D,E). These findings suggest that BDNF may play a crucial role in the NED of PCa, and its increased abundance was associated with the disease progression.

To investigate the potential role of BDNF in modulating immune cell behaviors within the TME, particularly focusing on its impact on immune cell infiltration in both treatment‐naïve and ADT‐treated prostate tumors, we conducted a macrophage polarization assessment. The human monocyte cell line THP‐1 was used for culture in PMA‐containing medium. M2 polarization, also known as alternative activation, represents a phenotype associated with anti‐inflammatory and immunosuppressive responses [45]. Results revealed that PMA‐treated THP‐1 cells cultured with medium collected from ADT‐treated LNCaP cells induced higher M2 marker (*CD206*, *CD163*, and *ARG1*) expression compared to treatment‐naïve cells (Fig. 6F). However, expression of M2 markers was reduced in THP‐1 cells cultured using media collected from BDNF‐KD‐expressing LNCaP cells following ADT (Fig. 6F). These assays provide insights into the impact of BDNF on M2 macrophage cell polarization, elucidating the mechanisms of BDNF‐mediated modulation of the immunosuppressive response within PCa after ADT. To evaluate the prognostic significance of the BDNF gene expression in patients undergoing immunotherapy, we utilized an online survival analysis tool to explore the impact of BDNF expression on the effectiveness of anti‐PD1 or anti‐PDL1 therapy. Notably, according to the Kaplan–Meier plotter dataset analysis [29], patients with high BDNF expression demonstrated a significant increase in the overall survival rate following anti‐PD1 or anti‐PDL1 treatment compared to patients who received no treatment (Fig. 6G). These data suggest that patients with elevated BDNF expression may experience improved survival outcomes when treated with anti‐PD1 or anti‐PDL1. Intriguingly, our analysis of TCGA PCa dataset revealed a substantial increase in *BDNF* mRNA levels associated with high PDL1 expression, as determined by the Pearson correlation analysis (Fig. 6H), supporting BDNF possibly serving as a potential biomarker for guiding anti‐PD1 or anti‐PDL1 therapy. In summary, our findings provide insights into the role of BDNF and its association with LIF/LIFR/STAT3‐driven neuroendocrine and immune checkpoint markers expression in PCa cells, highlighting their potential as therapeutic targets for treating advanced PCa. Crosstalk between PCa cells and prostate stromal cells may enhance the LIF/LIFR/STAT3 signaling‐driven immunosuppressive TME and NED in PCa cells through upregulation of BDNF (Fig. 6I).

## 4 Discussion

Patients with CRPC often display NED characteristics [46]. However, the mechanisms linking this process to an immunosuppressive response remain unclear. We sought to characterize molecular mechanisms of how the immunosuppressive response affects NED and study correlations between immunosuppressive cytokine abundance and malignant progression of PCa. Our study investigated the role of LIF/LIFR/STAT3 signaling in driving BNDF expression and its association with PDL1 abundance and NED in PCa cells after ADT. We demonstrated that activation of BDNF through ADT or LIF treatment resulted in increased expression of PDL1, neuroendocrine, and stem cell markers. Conversely, inhibition of LIF/LIFR/STAT3 signaling abolished the effects of ADT on the expression of BDNF and those markers. We assessed the abundance of BDNF in sera of patients with metastatic CRPC and explored its correlation with LIF/LIFR/STAT3‐driven neuroendocrine and stem cell marker expression. Our study provides evidence supporting the notion that LIF/LIFR/STAT3 signaling is a critical mediator of BDNF and PDL1 abundance in the TME of ADT‐resistant PCa.

The impact on PDL1 abundance of LIF/LIFR/STAT3 signaling in human prostate stromal cells has not been explicitly studied. PDL1 plays a crucial role in immune regulation by interacting with its receptor, PD1, in immune cells, leading to suppression of the immune response [47]. Tumor cells often exploit the PDL1/PD1 interaction to evade immune surveillance and immune‐mediated destruction [48]. A previous article focuses on the immunohistochemical expression of PDL1 in human PCa samples [49]. The primary objective is to assess the prevalence of PDL1 expression in different PCa types. Generally, PDL1 expression was found to be higher in PCa tissues when compared to benign prostate tissues or hyperplasia [49]. Notably, stromal cells exhibited PDL1 expression in 69% of cases, and this stromal PDL1 expression was correlated with PDL1 expression in tumor cells [49]. This study indicates that the expression of PDL1 in PCa and stromal cells exhibits variability across studies, influenced by factors such as tumor type, antibody clones, scoring criteria, and specimen types. Our study investigated how the activation of LIF/LIFR/STAT3 signaling in human prostate stromal cells affects the abundance of PDL1 in tumor cells. The experiments using CM from human prostate stromal cells showed that activation of the LIF/LIFR/STAT3 pathway in human prostate stromal cells was associated with increased levels of neuroendocrine markers, stem cell markers and immune checkpoints in tumor cells. *LIFR*‐KD in prostate stromal cells significantly reduced the expression of these markers in PCa cells, indicating the role of LIF/LIFR/STAT3 signaling in promoting NED and immune checkpoint abundance through paracrine signaling. Furthermore, CM from *LIFR*‐KD prostate stromal cells suppressed cell proliferation, sphere formation, migration, and invasion of PCa cells, suggesting a regulatory role of LIF/LIFR/STAT3 signaling in tumor‐promoting properties of human prostate stromal cells. Immunosuppressive cytokines were shown to suppress antitumor immune responses, contributing to immune evasion by cancer cells and facilitating tumor progression [35, 36]. Our findings revealed a possible positive correlation between LIF/LIFR/STAT3 upregulation and increased immunosuppressive immune cell type infiltration and a negative correlation with pro‐inflammatory immune cell types. Our results suggest that LIF/LIFR/STAT3 activation contributes to the immunosuppressive TME in PCa by promoting the infiltration of immunosuppressive immune cells.

The BDNF is primarily known for its role in the development and function of the nervous system [50], while there is limited direct research on the specific connection between NED and BDNF induction in PCa cells. Our study uncovered a potential link between LIF/LIFR/STAT3 signaling and BDNF‐driven NED in PCa after ADT resistance. The RNA‐Seq analysis revealed significant upregulation of BDNF and LIF in ADT‐resistant PCa cells compared to parental cells. Treatment of PCa cells with CSS‐containing medium to mimic ADT or with the LIF protein resulted in increased BDNF, LIF, and LIFR expressions. Moreover, LIF protein treatment led to activation of STAT3 and increased expression of BDNF and PDL1, while inhibition of LIF/LIFR/STAT3 signaling abolished those effects. These findings suggest that BDNF expression is regulated by LIF/LIFR/STAT3 signaling in PCa cells, and BDNF may play a role in mediating the development of an immunosuppressive TME. Alternatively, the LIF/LIFR/STAT3 signaling might indirectly impact the induction of BDNF by potentially influencing other elements within the TME, such as immune cells or stromal cells, both of which are recognized sources of BDNF secretion [51]. Existing studies have contributed to a broader comprehension of the intricate roles played by neurotrophins, specifically proNGF and NGF, within the complex landscape of prostate cancer [52, 53]. Our results consist with these studies insights offer valuable perspectives that could guide the development of future therapeutic strategies in addressing the multifaceted nature of prostate cancer and its neurotrophin‐related dynamics.

## 5 Conclusions

Our study provides insights into how LIF/LIFR/STAT3 signaling contributes to the upregulation of BDNF expression in stromal cells, increasing immune checkpoint levels, supporting NED, and promoting resistance to ADT in PCa. LIF/LIFR/STAT3 pathway activation fosters an immunosuppressive TME by triggering the expression of PDL1 and facilitating the infiltration of immunosuppressive immune cells. Patients with abundant BDNF expression exhibited significantly enhanced survival outcomes while undergoing anti‐PD1 or anti‐PDL1 treatment. Targeting LIF/LIFR/STAT3 signaling or downstream mediators, such as BDNF, could be potential therapeutic strategies to overcome immunosuppression, inhibit NED, and enhance the efficacy of immune‐based therapies in PCa. Further studies are warranted to validate these findings and explore the clinical implications of targeting LIF/LIFR/STAT3 signaling in PCa treatment.

## Conflict of interest

The authors declare no conflict of interest.

## Author contributions

Y‐NL and Y‐CW provided the conception and supervision of the study. M‐KL, H‐LY, W‐HC, K‐CJ, H‐RL, Z‐QC, and W‐HW performed the experiments and data analysis. H‐LY constructed the databases and performed the statistical and computational analyses. W‐YC performed the histomorphometric analysis. WA‐K designed the work, drafted the manuscript, and reviewed it critically for important intellectual content. WA‐K, Y‐NL, and Y‐CW wrote, reviewed, and revised the manuscript. All authors contributed to the article and approved the submitted version.

## Supporting information


**Fig. S1.** The leukemia‐inhibitory factor (LIF)/LIF receptor (LIFR)/signal transducer and activator of transcription 3 (STAT3) pathway‐driven neuroendocrine differentiation (NED) associates with PDL1 expression in prostate cancer (PCa) after androgen deprivation therapy (ADT).
**Fig. S2.** Associations between leukemia‐inhibitory factor receptor (LIFR) expression and immune response in prostate cancer (PCa) tumor microenvironment (TME).
**Fig. S3.** Androgen‐deprivation therapy (ADT) promotes the activation of leukemia‐inhibitory factor (LIF)/LIF receptor (LIFR)/signal transducer and activator of transcription 3 (STAT3) signaling, which is linked to the induction of BDNF in prostate cancer (PCa) cells.
**Fig. S4.** The abundance of brain‐derived neurotrophic factor (BDNF) is regulated by leukemia‐inhibitory factor (LIF) and is associated with the neuroendocrine differentiation (NED) and aggressiveness of prostate cancer (PCa) cells.
**Fig. S5.** Chromatin immunoprecipitation (ChIP)‐sequencing analysis of detected DNA‐binding sites for signal transduction and activator of transcription 3 (STAT3) and STAT3 response elements (SREs) of the brain‐derived neurotrophic factor (BDNF) and programmed death ligand 1 (PDL1/CD274) genes.
**Table S1.** Construction primer sequences.
**Table S2.** Reverse transcription‐quantitative real‐time polymerase chain reaction (RT‐qPCR) primer sequences.
**Table S3.** Western blotting antibodies.
**Table S4.** Immunohistochemistry (IHC) staining antibodies.
**Table S5.** Chromatin immunoprecipitation (ChIP) antibodies and primer sequences.

## Data Availability

The raw data supporting the conclusions of this article will be made available by the authors without undue reservation.
