# Supplementary material for: Immunosuppressive role of BDNF in therapy‐induced neuroendocrine prostate cancer
Source: Mol Oncol. 2024 Feb 21;18(6):1665–86. doi: 10.1002/1878-0261.13614 (PMC11161734; doi:10.1002/1878-0261.13614)
Supplement: Supplementary file 1 — Fig. S1. The leukemia‐inhibitory factor (LIF)/LIF receptor (LIFR)/signal transducer and activator of transcription 3 (STAT3) pathway‐driven neuroendocrine differentiation (NED) associates with PDL1 expression in prostate cancer (PCa) after androgen deprivation therapy (ADT). Fig. S2. Associations between leukemia‐inhibitory factor receptor (LIFR) expression and immune response in prostate cancer (PCa) tumor microenvironment (TME). Fig. S3. Androgen‐deprivation therapy (ADT) promotes the activation of leukemia‐inhibitory factor (LIF)/LIF receptor (LIFR)/signal transducer and activator of transcription 3 (STAT3) signaling, which is linked to the induction of BDNF in prostate cancer (PCa) cells. Fig. S4. The abundance of brain‐derived neurotrophic factor (BDNF) is regulated by leukemia‐inhibitory factor (LIF) and is associated with the neuroendocrine differentiation (NED) and aggressiveness of prostate cancer (PCa) cells. Fig. S5. Chromatin immunoprecipitation (ChIP)‐sequencing analysis of detected DNA‐binding sites for signal transduction and activator of transcription 3 (STAT3) and STAT3 response elements (SREs) of the brain‐derived neurotrophic factor (BDNF) and programmed death ligand 1 (PDL1/CD274) genes. Table S1. Construction primer sequences. Table S2. Reverse transcription‐quantitative real‐time polymerase chain reaction (RT‐qPCR) primer sequences. Table S3. Western blotting antibodies. Table S4. Immunohistochemistry (IHC) staining antibodies. Table S5. Chromatin immunoprecipitation (ChIP) antibodies and primer sequences. [file MOL2-18-1665-s001.docx]

**Supplementary File**

**
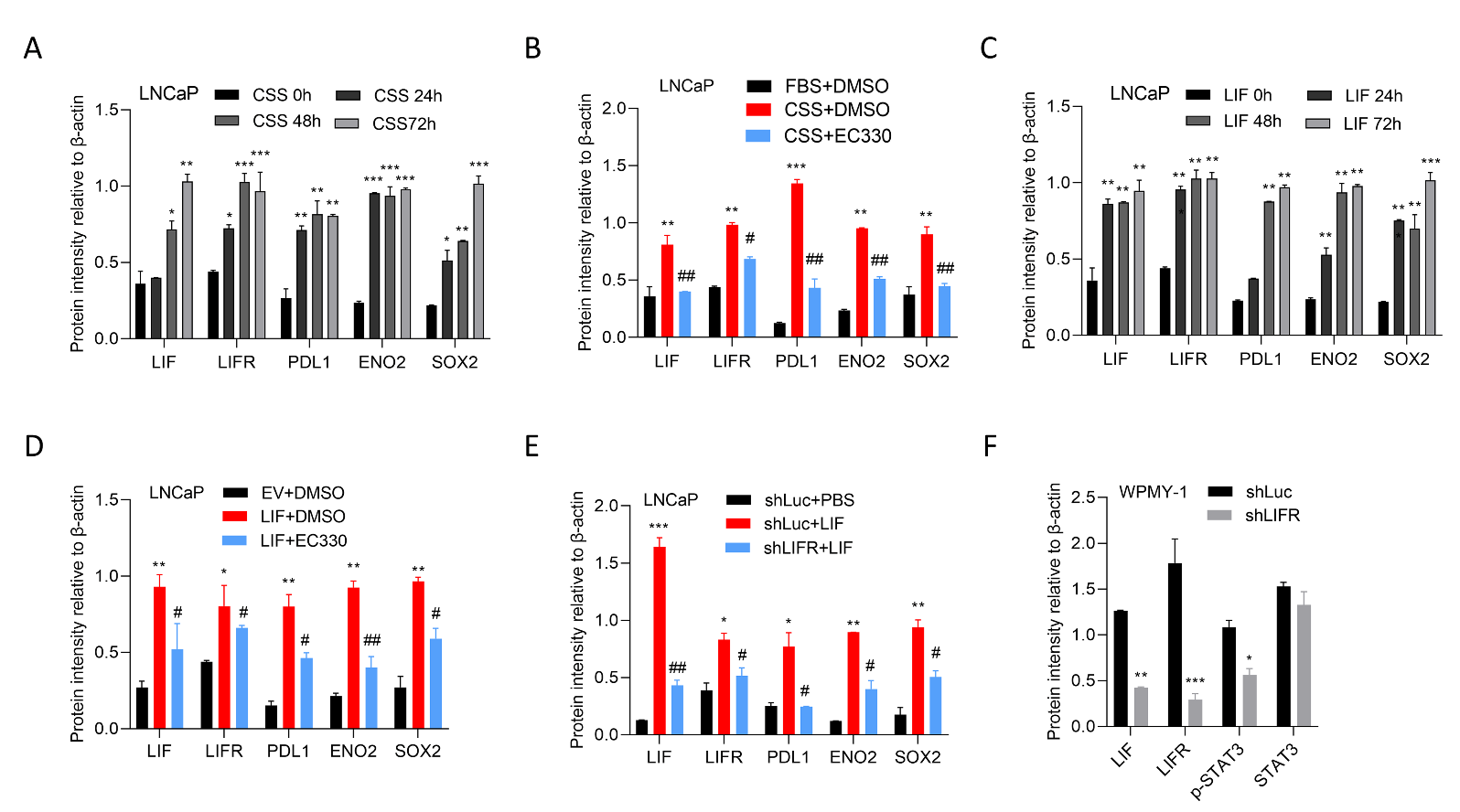
Supplementary Figures**

**Sup. Fig. S1. The leukemia-inhibitory factor (LIF)/LIF receptor (LIFR)/signal transducer and activator of transcription 3 (STAT3) pathway-driven neuroendocrine differentiation (NED) associates with PDL1 expression in prostate cancer (PCa) after androgen deprivation therapy (ADT). A** Relative protein intensity of LIF, LIFR, PDL1, ENO2, and SOX2 in LNCaP cells cultured in charcoal-stripped serum (CSS)-containing medium for 0~72 h. Asterisks vs. CSS 0 h. **B** Relative protein intensity of LIF, LIFR, PDL1, ENO2, and SOX2 in LNCaP cells treated with CSS-containing medium, followed by treatment with DMSO or 35 nM EC330 for 24 h. Asterisks vs. FBS+DMSO; hashtags vs. CSS+DMSO. **C** Relative protein intensity of LIF, LIFR, PDL1, ENO2, and SOX2 in LNCaP cells treated with 100 ng/ml of the LIF recombinant protein for 0~72 h. Asterisks vs. LIF 0 h. **D** Relative protein intensity of LIF, LIFR, PDL1, ENO2, and SOX2 in LNCaP cells expressing an empty vector (EV) or *LIF* cDNA vector, followed by treatment with 35 nM EC330 for 24 h. Asterisks vs. EV+DMSO. **E** Relative protein intensity of LIF, LIFR, PDL1, ENO2, and SOX2 in LNCaP cells stably transfected with Luc or *LIFR* shRNA, followed by treatment with PBS or 100 ng/ml of the LIF recombinant protein for 48 h. Asterisks vs. shLuc+PBS. **F** Relative protein intensity of LIF, LIFR, p-STAT3, and STAT3 in WPMY-1 cells stably transfected with Luc or *LIFR* shRNA. Asterisks vs. shLuc. Protein intensities were measured by ImageJ software and determined from three independent experiments, as determined by a one-way ANOVA and t-test. * *p* < 0.05, ** *p* < 0.01, *** *p* < 0.005, # *p* < 0.05, ## *p* < 0.01.

**
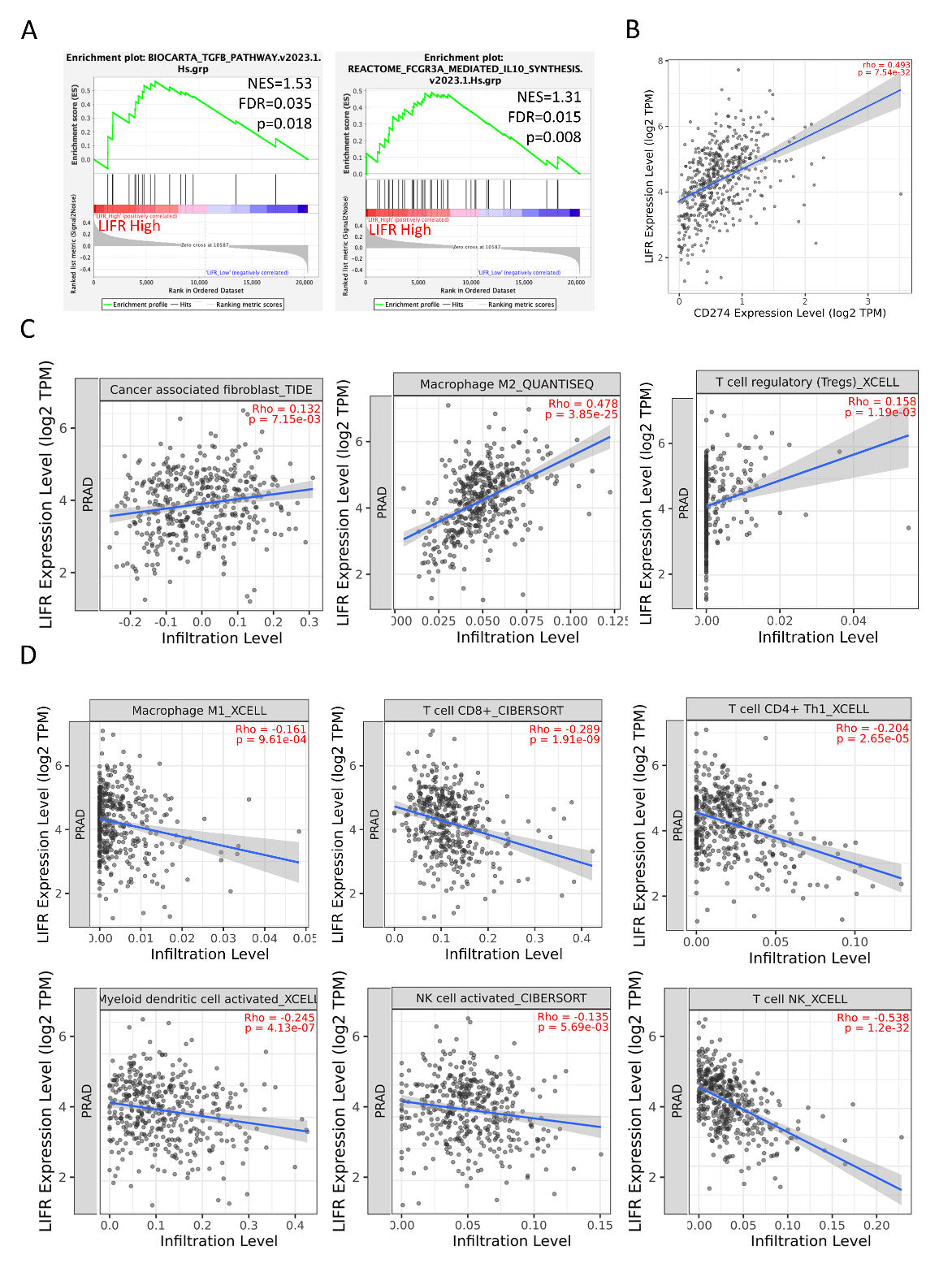
**

**Sup. Fig. S2. Associations between leukemia-inhibitory factor receptor (LIFR) expression and immune response in prostate cancer (PCa) tumor microenvironment (TME).** **A** Gene Set Enrichment Analysis (GSEA) of the TCGA PCa dataset revealed a positive association between high *LIFR* mRNA abundance in PCa samples and the upregulation of gene signatures related to immunosuppressive cytokine responsiveness, including transforming growth factor- ß (TGF-ß) and interleukin (IL)-10 (BIOCARTA and REACTOME). NES, normalized enrichment score; FDR, false discovery rate. **B** Correlation analyses of *LIFR* and programmed death ligand 1 (*PDL1*) mRNA levels in prostate tissue samples from TCGA PCa datasets. Correlation coefficients (*R*^2^) and *p* values were determined by correlation XY analyses in GraphPad Prism. *R*=0.493; *p*=7.54e-32. **C** Spearman’s values showed positive relationships between LIFR and immune infiltration cells (cancer-associated fibroblasts, M2 macrophages, and regulatory T (Treg) cells) in prostate adenocarcinoma (PRAD) using TIMER2 database by a purity-adjusted Spearman's rho analysis. **D** Spearman’s values showed negative relationships between LIFR and immune infiltrating cells (M1 macrophage, CD8+ T cells, CD4+ T cells, myeloid dendritic cells, natural killer (NK) cells, and NK T cells) in TIMER2 database by a purity-adjusted Spearman's rho analysis.

**
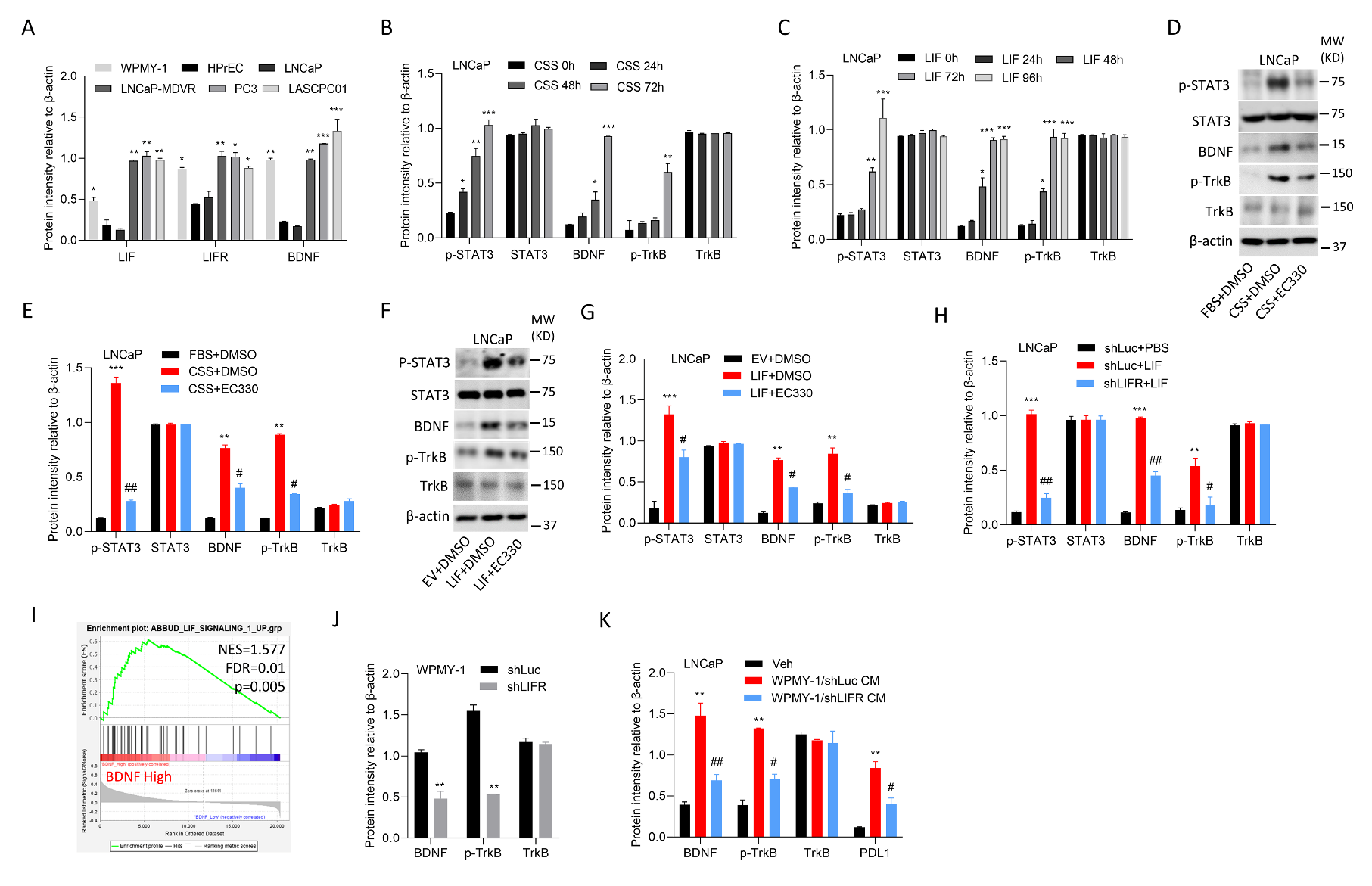
**

**Sup. Fig. S3. Androgen-deprivation therapy (ADT) promotes the activation of** **leukemia-inhibitory factor (LIF)/LIF receptor (LIFR)/signal transducer and activator of transcription 3 (STAT3) signaling, which is linked to the induction of BDNF in prostate cancer (PCa) cells.** **A** Relative protein intensity of LIF, LIFR, and BDNF in prostate stromal cells (WPMY-1), normal prostate epithelial cells (HPrEC), prostate adenocarcinoma cells (LNCaP and PC3), MDV3100-resistant cells (LNCaP-MDVR), and NEPC-like (LASCPC01) cells. Asterisks vs. HPrEC. **B** Relative protein intensity of p-STAT3, STAT3, BDNF, p-TrkB, and TrkB in LNCaP cells cultured in charcoal-stripped serum (CSS)-containing medium for 0~72 h. Asterisks vs. CSS 0 h. **C** Relative protein intensity of p-STAT3, STAT3, BDNF, p-TrkB, and TrkB in LNCaP cells treated with 100 ng/ml of the LIF recombinant protein for 0~96 h. Asterisks vs. LIF 0 h. **D, E** Protein levels and relative intensity of p-STAT3, STAT3, BDNF, p-TrkB, and TrkB in LNCaP cells were treated with CSS-containing medium for 5 days, followed by treatment with 35 nM EC330 for 24 h. Asterisks vs. FBS+DMSO; hashtags vs. CSS+DMSO. **F, G** Protein levels and relative intensity of p-STAT3, STAT3, BDNF, p-TrkB, and TrkB in LNCaP cells stably expressing an empty vector (EV) or *LIF* cDNA vector, followed by treatment with DMSO or 35 nM EC330 for 24 h. Asterisks vs. EV+DMSO; hashtags vs. LIF+DMSO. **H** Relative protein intensity of p-STAT3, STAT3, BDNF, p-TrkB, and TrkB in LNCaP cells stably transfected with a non-target control (Luc) or *LIFR* shRNA vector, followed by treatment with phosphate-buffered saline (PBS) or 100 ng/ml of the LIF recombinant protein for 48 h. Asterisks vs. shLuc+PBS; hashtags vs. shLuc+LIF. **I** Gene set enrichment analysis (GSEA) of TCGA PCa dataset showing that high abundances of *BDNF* mRNAs in PCa samples were positively associated with LIF signaling (Appud) responsiveness gene signatures. NES, normalized enrichment score; FDR, false discovery rate. **J** Relative protein intensity of BDNF, p-TrkB, and TrkB in WPMY-1 cells stably transfected with Luc or *LIFR* shRNA. Asterisks vs. shLuc. **K** Relative protein intensity of BDNF, p-TrkB, TrkB, and PDL1 in LNCaP cells cultured with conditioned medium (CM) collected from Luc or *LIFR* shRNA-expressing WPMY-1 cells for 48 h. Asterisks vs. Veh; hashtags vs. WPMY-1/shLuc CM. Protein intensities were measured by ImageJ software and determined from three independent experiments, as determined by a one-way ANOVA and t-test. * *p* < 0.05, ** *p* < 0.01, *** *p* < 0.005, # *p* < 0.05, ## *p* < 0.01.

**
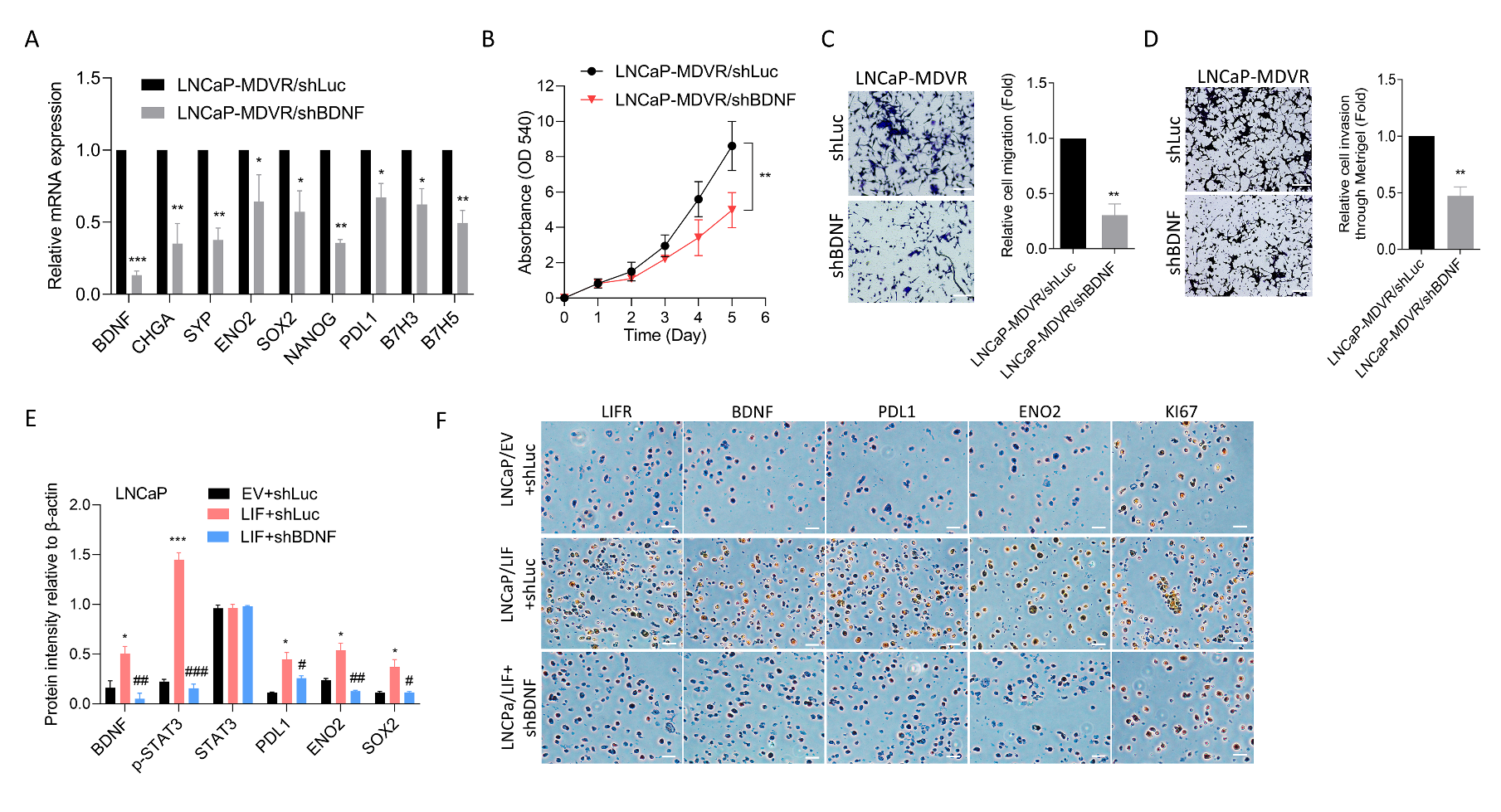
**

**Sup. Fig. S4. The abundance of** **brain-derived neurotrophic factor (BDNF) is regulated by** **leukemia-inhibitory factor (LIF) and is associated with the neuroendocrine differentiation (NED) and aggressiveness of prostate cancer (PCa) cells. A** Relative mRNA levels of *BDNF*, neuroendocrine (*CHGA, SYP*, and *ENO2*), stem cell (*SOX2* and *NANOG*), and immune checkpoint (*PDL1, B7H3*, and *B7H5*) markers in LNCaP-MDVR cells stably transfected with a non-target control (Luc) vector or *BDNF* shRNA vector. Asterisks vs. shLuc, by a one-way ANOVA and t-test. **B** Cell proliferation assays of LNCaP-MDVR cells stably transfected with Luc or *BDNF* shRNA vector. *n*=8 per group. Asterisks vs. shLuc, as determined by a one-way ANOVA and t-test. **C, D** Relative cell migration (**C**) and invasion through Matrigel (**D**) were assessed in LNCaP-MDVR cells stably transfected with Luc or *BDNF* shRNA vector for 12 h. Scale bars represent 100 µm. Asterisks vs. shLuc, as determined by a one-way ANOVA and t-test. **E** Relative protein intensity of BDNF, p-STAT3, STAT3, PDL1, ENO2, and SOX2 in LNCaP cells stably transfected with the empty vector (EV) or *LIF* cDNA vector, followed by stable transfection with Luc or *BDNF* shRNA vector. Asterisks vs. EV+shLuc; hashtags vs. LIF+shLuc. **F** Immunohistochemical (IHC) staining on cell pellets of LNCaP/EV+shLuc, LNCaP/LIF+shLuc, and LNCaP/LIF+shBDNF cells with LIFR, BDNF, PDL1, ENO2, and KI67 antibodies. Protein intensities were measured by ImageJ software and determined from three independent experiments, as determined by a one-way ANOVA and t-test. Quantification of relative mRNA levels is presented as the mean ± SEM from three biological replicates. * *p* < 0.05, ** *p* < 0.01, *** *p* < 0.005, # *p* < 0.05, ## *p* < 0.01, ### *p* < 0.005.

**
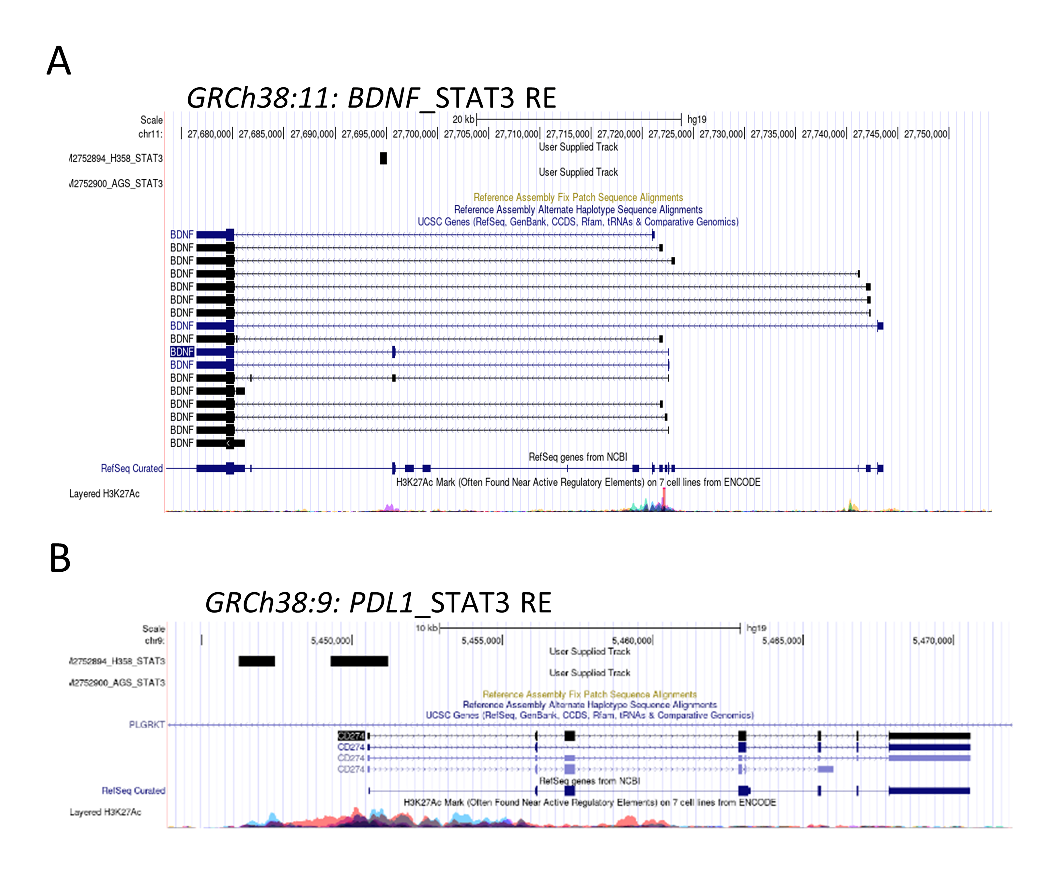
**

**Sup. Fig. S5. Chromatin immunoprecipitation (ChIP)-sequencing analysis of detected DNA-binding sites for signal transduction and activator of transcription 3 (STAT3) and STAT3 response elements (SREs) of the brain-derived neurotrophic factor (*BDNF*) and programmed death ligand 1 (*PDL1/CD274*) genes. A, B** ChIP-sequencing data were downloaded from the Gene Expression Omnibus (GEO) (GSM2752894 and GSM2752900) to investigate the binding potential of STAT3 to the SRE of the *BDNF* (A) and *PDL1* (B) genes by Genome Brower (UCSC).

**Supplementary Tables**

**Supplementary Table S1. Constructions primer sequences.** Primer sequences of the wild-type (WT) and mutant (M) signal transduction and activator of transcription 3 (STAT3)-binding elements of human brain-derived neurotrophic factor (*BDNF*) or programmed death ligand 1 (*PDL1*) regulatory sequence reporters are listed 5’-3’. M, mutant; F, forward; R, reverse.

| Promoter reporter | Sequence |
| --- | --- |
| *BDNF*/SRE1_WT F | TCTACAGATGGTTTGGATGTATGG |
| *BDNF*/SRE1_WT R | GCTCTCACCTGGAAGTTTCTAAT |
| *BDNF*/SRE2_WT F | TCAATGGGTTCACTGATCTTTCC |
| *BDNF*/SRE2_WT R | GCTGGCCTACTCACTTGTTAAT |
| *BDNF*/SRE3_WT F | CACACACACACACAGAGAGAA |
| *BDNF*/SRE3_WT R | AACAATGTGTCTGGTAAGGAGAA |
| *BDNF*/SRE1_M F | ATTAGAGCGTGGCTAG AAAAGAGGTGCAGCCTTG |
| *BDNF*/SRE1_M R | CTAGCCACGCTCTAATGCAAGTACTTGGATA |
| *BDNF*/SRE2_M F | GGCTCAGACATGATCCATTTTCTAAAAGAACTTACCTTTCA |
| *BDNF*/SRE2_M R | tggatcatgtctgagcccccataat |
| *BDNF*/SRE3_M F | GAAAAAGCCAAGGAGTGAAGAACCTTAGAGAACTGG |
| *BDNF*/SRE3_M R | cactccttggcttttctggctaata |
| *PDL1*/SRE1_WT F | CCTGAAAGAGTATCTCTCAATTGT |
| *PDL1*/SRE1_WT R | CCCATGCCACAGGTACTTTA |
| *PDL1*/SRE2_WT F | GCAGAAGGAAGGATGGTACTG |
| *PDL1*/SRE2_WT R | CTGCCTGGGATCCAAACTT |
| *PDL1*/SRE3_WT F | GCCTGTAATATCAGCACTTTGG |
| *PDL1*/SRE3_WT R | CCATCCCGAGCTACATCTTT |
| *PDL1*/SRE1_M F | AAATATTTACCACAGAGTG TTCTTAGACTCCCACAGCAT |
| *PDL1*/SRE1_M R | CACTCTGTGGTAAATATTTTAAATCTAGGG |
| *PDL1*/SRE2_M F | GAACTCTTGGCTCATTTTTTCTTGCAAATATTAAATCAC |
| *PDL1*/SRE2_M R | AAAATGAGCCAAGAGTTCTTTGCAAGTACA |
| *PDL1*/SRE3_M F | AACCCAAAGCTTTCAAAAGTTACTTCTTAACCCTCAC |
| *PDL1*/SRE3_M R | TTGAAAGCTTTGGGTTAGTGAATGGGCCCAA |

**Supplementary Table S2. Reverse transcription-quantitative real-time polymerase chain reaction (RT-qPCR) primer sequences.** Sequences of the primers used in real-time qPCR assays in this study are listed 5’-3’. F, forward; R, reverse; m, murine.

| Gene | Primer sequence | Gene | Primer sequence |
| --- | --- | --- | --- |
| *LIF* F | CGGGACCAGAAGATCCTCAA | *LIF* R | ACAGCCCAGCTTCTTCTTCT |
| *LIFR* F | GCTGTCATTGTTGGAGTGGTG | *LIFR* R | CTGCTTCCCTCACAGACACT |
| *PDL1* F | GGTAAGACCACCACCACCAA | *PDL1* R | TTGGAGGATGTGCCAGAGG |
| *CHGA* F | ACTGAAGGAGCTCCAAGAC | *CHGA* R | TCTGCCTCCTTGGAATCCTC |
| *ENO2* F | TCAGGGACTACCTGTGGTCT | *ENO2* R | TTCCACTGCCGCTCAATAC |
| *SYP* F | GGCTTTGTGAAGGTGCTGC | *SYP* R | CACTCTCGGTCTTGTTGGCA |
| *SOX2* F | ACCAGCTCGCAGACCTACAT | *SOX2* R | CCTGCTGCGAGTAGGACAT |
| *NANOG* F | AAGGTCCCGGTCAAGAAACAG | *NANOG* R | CTTCTGCGTCACACCATTGC |
| *PDL1* F | ACAGTCATCTGTCTGGACGC | *PDL1* R | TGTCCTCGGATGGCTACAGT |
| *B7H3* F | CTGTCTGTCTCATTGCACTGCTG | *B7H3* F | TTTGCTGTCAGAGTGTTTCAGAGGC |
| *B7H5* F | GATTTTGGTGCCCTCTGCGATTTTG | *B7H5* R | TCTTTCTTGTTGGGCTCCATCAGC |
| *CD206* F | CGAGGAAGAGGTTCGGTTCAC | *CD206* R | CAATCCCGGTTCTCATGGCA |
| *CD163* F | CCAGAAGGAACTTGTAGCCACAG | *CD163* R | CTATGTCCCAGTGAGAGTTACAGAG |
| *ARG1* F | TCATCTGGGTGGATGCTCACAC | *ARG1* R | AGAATCCTGGCACATCGGGAATC |
| *18S* F | CATGGCCGTTCTTAGTTGGTGG | *18S* R | CGCTGAGCCAGTCAGTGTAG |

**Supplementary Table S3.** **Western blotting antibodies.** The source and dilution of each antibody used for Western blotting in this study are listed. cat. no., catalog number.

| Primary antibody | Clonality | Source  (cat. no.) | Dilution | Secondary antibody | Source | Dilution |
| --- | --- | --- | --- | --- | --- | --- |
| LIF | Polyclonal | Abcam  (ab113262) | 1:1000 | anti-rabbit IgG | Jackson Labs | 1:5000 |
| LIFR | Polyclonal | Abcam (ab101228) | 1:1000 | anti-rabbit IgG | Jackson Labs | 1:5000 |
| ENO2 | Monoclonal | Santa Cruz  (sc-21738) | 1:100 | anti-mouse IgG | Jackson Labs | 1:5000 |
| PDL1 | Monoclonal | Proteintech (66248-1-lg) | 1:5000 | anti-mouse IgG | Jackson Labs | 1:5000 |
| SOX2 | Polyclonal | Cell signaling (2748) | 1:1000 | anti-rabbit IgG | Jackson Labs | 1:5000 |
| p-STAT3 | Polyclonal | Cell signaling (9145S) | 1:1000 | anti-rabbit IgG | Jackson Labs | 1:5000 |
| STAT3 | Monoclonal | Cell signaling (9139S) | 1:1000 | anti-mouse IgG | Jackson Labs | 1:5000 |
| BDNF | Monoclonal | Abcam (ab108319) | 1:1000 | anti-rabbit IgG | Jackson Labs | 1:5000 |
| p-TrkB (Tyr816) | Polyclonal | Sigma-Aldrich  (ABN1381) | 1:1000 | anti-rabbit IgG | Jackson Labs | 1:5000 |
| TrkB | Polyclonal | Sigma-Aldrich  (SAB4300702) | 1:1000 | anti-rabbit IgG | Jackson Labs | 1:5000 |
| β-actin | Polyclonal | GeneTex (GTX109639) | 1:1000 | anti-rabbit IgG | Jackson Labs | 1:20000 |

**Supplementary Table S4: Immunohistochemistry (IHC) staining antibodies.** The source and dilution of each antibody as well as the method of antigen retrieval used for the IHC assays in this study are listed. HRP, horseradish peroxidase-conjugated. cat. no., catalog number.

| Primary antibody | Clonality | Source  (cat. no.) | Dilution | Antigen retrieval | Secondary antibody | Source  (cat. no.) |
| --- | --- | --- | --- | --- | --- | --- |
| LIFR | Polyclonal | Abcam (ab101228) | 1:100 | Autoclave | HRP anti-mouse | DAKO E0433 |
| BDNF | Monoclonal | Abcam (ab108319) | 1:100 | Autoclave | HRP anti-ribbit | DAKO E0433 |
| ENO2 | Monoclonal | Santa Cruz  (sc-21738) | 1:100 | Autoclave | HRP anti-mouse | DAKO E0433 |
| PDL1 | Monoclonal | Proteintech (66248-1-lg) | 1:1000 | Autoclave | HRP anti-rabbit | DAKO E0432 |
| KI67 | Polyclonal | Abcam (ab15580) | 1:200 | Autoclave | HRP anti-rabbit | DAKO E0432 |

**Supplementary Table S5.** **Chromatin immunoprecipitation (ChIP) antibodies and primer sequences.** The source and dilution of each antibody and the sequences (5’-3’) of each primer used for ChIP in this study are listed. cat. no., catalog number; p, phosphorylated.

| ChIP antibodies | | | | |
| --- | --- | --- | --- | --- |
| Primary antibody | Species | Clonality | Source (cat. no.) | Dilution |
| p-STAT3 | Rabbit | Monoclonal | Abcam (ab108341) | 1:50 |
| Acety-H3 | Rabbit | Monoclonal | Novus (NB300-221) | 1:100 |
| IgG | Rabbit |  | Santa Cruz (sc-2027) | 1:50 |
| ChIP primers | | | | |
| Site | Sequence | | | |
| *BDNF*/SRE1 F | TCTACAGATGGTTTGGATGTATGG | | | |
| *BDNF*/SRE1 R | GCTCTCACCTGGAAGTTTCTAAT | | | |
| *BDNF*/SRE2 F | TCAATGGGTTCACTGATCTTTCC | | | |
| *BDNF*/SRE2 R | GCTGGCCTACTCACTTGTTAAT | | | |
| *BDNF*/SRE3 F | CACACACACACACAGAGAGAA | | | |
| *BDNF*/SRE3 R | AACAATGTGTCTGGTAAGGAGAA | | | |
| *PDL1*/SRE1 F | CCTGAAAGAGTATCTCTCAATTGT | | | |
| *PDL1*/SRE1 R | CCCATGCCACAGGTACTTTA | | | |
| *PDL1*/SRE2 F | GCAGAAGGAAGGATGGTACTG | | | |
| *PDL1*/SRE2 R | CTGCCTGGGATCCAAACTT | | | |
| *PDL1*/SRE3 F | GCCTGTAATATCAGCACTTTGG | | | |
| *PDL1*/SRE3 R | CCATCCCGAGCTACATCTTT | | | |
